# Active receptor tyrosine kinases, but not Brachyury, are sufficient to trigger chordoma in zebrafish

**DOI:** 10.1242/dmm.039545

**Published:** 2019-07-16

**Authors:** Gianluca D'Agati, Elena María Cabello, Karl Frontzek, Elisabeth J. Rushing, Robin Klemm, Mark D. Robinson, Richard M. White, Christian Mosimann, Alexa Burger

**Affiliations:** 1 Institute of Molecular Life Sciences, University of Zürich, 8057 Zürich, Switzerland; 2 Institute of Neuropathology, University Hospital Zürich, 8091 Zürich, Switzerland; 3 SIB Swiss Institute of Bioinformatics, University of Zürich, 8057 Zürich, Switzerland; 4 Cancer Biology & Genetics, Memorial Sloan Kettering Cancer Center, New York, NY 10065, USA; 5 Department of Medicine, Memorial Sloan Kettering Cancer Center, New York, NY 10065, USA

**Keywords:** Notochord, *TBXT*, RTK, Cancer, *Danio rerio*, *In vivo* models

## Abstract

The aberrant activation of developmental processes triggers diverse cancer types. Chordoma is a rare, aggressive tumor arising from transformed notochord remnants. Several potentially oncogenic factors have been found to be deregulated in chordoma, yet causation remains uncertain. In particular, sustained expression of *TBXT* – encoding the notochord regulator protein brachyury – is hypothesized as a key driver of chordoma, yet experimental evidence is absent. Here, we employ a zebrafish chordoma model to identify the notochord-transforming potential of implicated genes *in vivo*. We find that Brachyury, including a form with augmented transcriptional activity, is insufficient to initiate notochord hyperplasia. In contrast, the chordoma-implicated receptor tyrosine kinases (RTKs) EGFR and Kdr/VEGFR2 are sufficient to transform notochord cells. Aberrant activation of RTK/Ras signaling attenuates processes required for notochord differentiation, including the unfolded protein response and endoplasmic reticulum stress pathways. Our results provide the first *in vivo* evidence against a tumor-initiating potential of Brachyury in the notochord, and imply activated RTK signaling as a possible initiating event in chordoma. Furthermore, our work points at modulating endoplasmic reticulum and protein stress pathways as possible therapeutic avenues against chordoma.

## Abstract (precis)

**Summary:** An injection-based chordoma model in zebrafish shows that the hypothesized chordoma oncogene brachyury is insufficient, whereas EGFR and VEGFR2 are sufficient, to trigger notochord hyperplasia in our model.

## INTRODUCTION

Different genetic lesions can result in the malignant transformation of individual cells, ultimately leading to cancer. In particular, the reactivation or overexpression of developmental transcription factor genes has been repeatedly implicated in a variety of tumors, including *TAL1* (*SCL*) and *RUNX1* in leukemia (Porcher et al., 2017; Sood et al., 2017), *SOX10* and *MITF* in melanoma (Johannessen et al., 2013; Kaufman et al., 2016) and *GLI1* in glioma (Clement et al., 2007). Constitutive activation of developmental signaling pathways seems even more common, in particular through activating mutations and amplifications of receptor tyrosine kinase (RTK) genes including EGFR, FGFR or VEGFR family genes (Sharma and Settleman, 2007). The permissive and instructive functions of these factors deployed in the embryo, when reactivated or maintained unchecked in adult tissues, endow transformed cells with malignant properties of hyperproliferation, stemness and tissue invasion (Hanahan and Weinberg, 2011). Nevertheless, the relationship between developmental regulators and the maintenance of lineage identity for a given cancer's cell of origin remains challenging to decipher.

Chordoma is a rare, slow-growing tumor typically occurring at the base of the skull or in sacrococcygeal regions along the spine (OMIM #215400). Chordoma is thought to result from malignant transformations of remnant cells of the embryonic notochord, a cartilaginous structure of mesodermal origin that supports embryo axis formation and spinal column formation before regressing during development (Yakkioui et al., 2014). Despite its proposed origin from embryonic notochord cells, chordoma is typically first diagnosed in adults between the ages of 40 and 70, with an annual incidence of one case per million per year (Smoll et al., 2013). Surgical removal of the tumor remains the most effective therapeutic option. Nevertheless, owing to the deep tissue localization of chordomas, surgery of the strongly radio- and chemoresistant tumor is highly delicate or, in individual cases, even impossible. In addition, first treatment frequently results in reoccurring local tumors and possible metastases even after seemingly successful removal of the initial lesion. No proven systemic therapies are available for patients with reoccurring or non-resectable disease, nor against the ultimately fatal distant metastases (Walcott et al., 2012).

To date, no single molecular pathway can be assigned as a fundamental chordoma-driving mechanism. Genomic sequencing of patient chordoma samples continues to uncover mutated genomic loci including *PTEN* (Choy et al., 2014), the tuberous sclerosis complex (TSC) genes (Han et al., 2009), *TP53* (Pallini et al., 2003) and *BCL6* (Rinner et al., 2013). Furthermore, chordoma samples repeatedly show increased expression of, amplifications in or possibly activating mutations in several RTK genes, including *EGFR* and the chromosome 4q12 genes *VEGFR2* (*KDR*), *KIT* and *PDGFRA* that relay their activity through Ras (Akhavan-Sigari et al., 2014a,b,c; de Castro et al., 2013; Fischer et al., 2015; Miettinen et al., 2012; Tamborini et al., 2006). Emphasizing the potential significance of reoccurring RTK involvement, EGF pathway inhibitors have been shown to curb the growth of chordoma cell lines and xenografts (Scheipl et al., 2016). Clinical trials have been performed using a variety of RTK inhibitors, including imatinib, sunitinib and EGFR inhibitors, as well as with the mTOR inhibitor rapamycin combined with imatinib (Walcott et al., 2012).

A seemingly consistent feature of chordoma is the expression of the T-box transcription factor T gene *T* (also known as *TBXT*; Presneau et al., 2011), which encodes brachyury protein. During development, *TBXT* influences individual mesodermal fates and is an evolutionarily conserved regulator of the notochord (Cunliffe and Ingham, 1999; Kispert and Herrmann, 1993; Nibu et al., 2013). Intriguingly, the genomic locus encoding for *TBXT* has been found to be duplicated or further amplified in familial and sporadic chordoma cases (Hallor et al., 2008; Presneau et al., 2011; Yang et al., 2009). This data, together with detectable brachyury gene and protein expression in the vast majority of chordomas, points to a tumor origin from transformed remnant notochord cells. The transient nature of the notochord, which disappears before birth, and persistence of notochord cells in chordoma suggest that malignant transformation could depend on an early developmental pathway that maintains a notochord or early mesodermal program in remnant notochord cells. The expression of *TBXT* is also increasingly reported in other cancers of the lung, small intestine, stomach, kidney, bladder, uterus, ovary and testis: its expression correlates with epithelial-to-mesenchymal transition (EMT), maintenance of an undifferentiated state and the resistance of lung cancer cells to EGFR inhibition (Roselli et al., 2012). It has been suggested that brachyury inhibits the cell cycle by downregulating cyclin D (*CCND1*), *RB* (*RB1*) and *CDKN1A* (*P21*), ultimately decreasing the susceptibility of tumor cells to cytotoxic therapies (Huang et al., 2013). Nonetheless, recent notochord-specific knockdown of brachyury (*T*) in mouse revealed that its activity is dispensable for notochord proliferation and EMT, questioning the potency of brachyury misexpression or overexpression alone to mediate chordoma formation (Zhu et al., 2016). Consequently, the influence of brachyury expression on chordoma initiation and tumor maintenance *in vivo* remains speculative, as are the pathways that maintain the notochord in an oncogenic progenitor state.

The lack of a unifying mechanism leading to chordoma formation necessitates the development of animal models that recapitulate key aspects of the disease. Given the proposed developmental origins of chordoma, the zebrafish offers a unique opportunity to dissect the mechanisms of tumor initiation and progression. Controlled by regulators including Brachyury that drive deeply conserved notochord-forming mechanisms across vertebrates, the early notochord is principally formed by central vacuolated cells that provide hydraulic stability. In zebrafish, *TBXT* is present as two paralogs, *tbxta* and *tbxtb*, of which *tbxta* mutants have been initially described as *no tail* (*ntl* or *ntla*) owing to their prominent loss of the notochord and tail (Halpern et al., 1993; Schulte-Merker et al., 1994). Notch signaling-dependent differentiation results in an epithelial layer of outer sheath cells (Dale and Topczewski, 2011; Yamamoto et al., 2010). The sheath cells surround the vacuole cells and secrete an extracellular matrix (ECM) composed of collagens, laminins and proteoglycans that encapsulate the notochord. Work in medaka has established that endoplasmic reticulum (ER) stress occurs physiologically during this process, which requires the unfolded protein response (UPR) transducers Atf6 and Creb3l2 for the proper alignment of the notochord cells and for the export of type II collagen (Ishikawa et al., 2013, 2017a). Notochord formation in *Xenopus* has also been linked to the progressive activation of UPR via Xbp1 and Creb3l2 to drive its differentiation (Tanegashima et al., 2009). Subsequently, the notochord ossifies to form the spine segments, whereas remnant notochord cells turn into the gel-like nucleus pulposus inside the intervertebral discs of the spinal column (Grotmol et al., 2003, 2005; LLeras Forero et al., 2018; Pogoda et al., 2018; Wopat et al., 2018).

We recently established in zebrafish the first animal proxy for chordoma onset based on notochord-specific expression of *HRAS^V12^*, which recapitulates oncogenic RTK/Ras pathway activation (Burger et al., 2014). Our model uses the bimodal Gal4/*UAS* system, in which a notochord-specific transgene expresses the Gal4 transcription factor that drives a separate candidate transgene with upstream activating sites (*UAS*). With virtually 100% of animals affected as early as 2-3 days post-fertilization (dpf), *HRAS^V12^*-expressing embryos rapidly develop prominent notochord hyperplasia that shares key histological features with human chordoma samples (Burger et al., 2014).

Here, we extended this *in vivo* platform to test the potency of chordoma-implicated factors in driving notochord hyperplasia. We generated *col2a1aR2:KalTA4* transgenic zebrafish as a basis to establish a robust mosaic assay for injection-based candidate gene expression with fluorescent tags in the developing notochord. The *col2a1aR2*-driven transient mosaic expression of *HRAS^V12^* triggered chordoma-like notochord hyperplasia akin to stable genetic insertions. Notably, notochord-focused overexpression of human *TBXT* or zebrafish *tbxta*/*tbxtb*, including a version with augmented transcriptional activity, failed to cause notochord hyperplasia in the observed timeframe of 5 dpf. By contrast, overexpression of the chordoma-implicated RTK genes *EGFR* and *kdr* (*vegfr2*) potently induced a chordoma phenotype. Transcriptome sequencing and cellular ultrastructure analysis revealed that Ras-mediated RTK signaling drives excessive secretory pathway activity, ECM build up and suppression of the UPR in transformed notochord sheath cells. These processes hinder the transformed sheath cells from further differentiation, yet ultimately trap the hyperproliferating mass with its accumulating ECM. Taken together, our data indicate that brachyury per se might be insufficient to initiate chordoma and rather reflects the maintained notochord lineage identity of the transformed cells. Instead, our results suggest that aberrant RTK signaling, possibly through the activation of repeatedly chordoma-implicated RTK genes (Akhavan-Sigari et al., 2014a,b,c; de Castro et al., 2013; Fischer et al., 2015; Miettinen et al., 2012; Tamborini et al., 2006), and a maintained early notochord program involving ER stress and the UPR, present potent initial events towards chordoma formation.

## RESULTS

### Sheath cell-directed oncogene expression drives chordoma formation in zebrafish

**Fig. 1.**
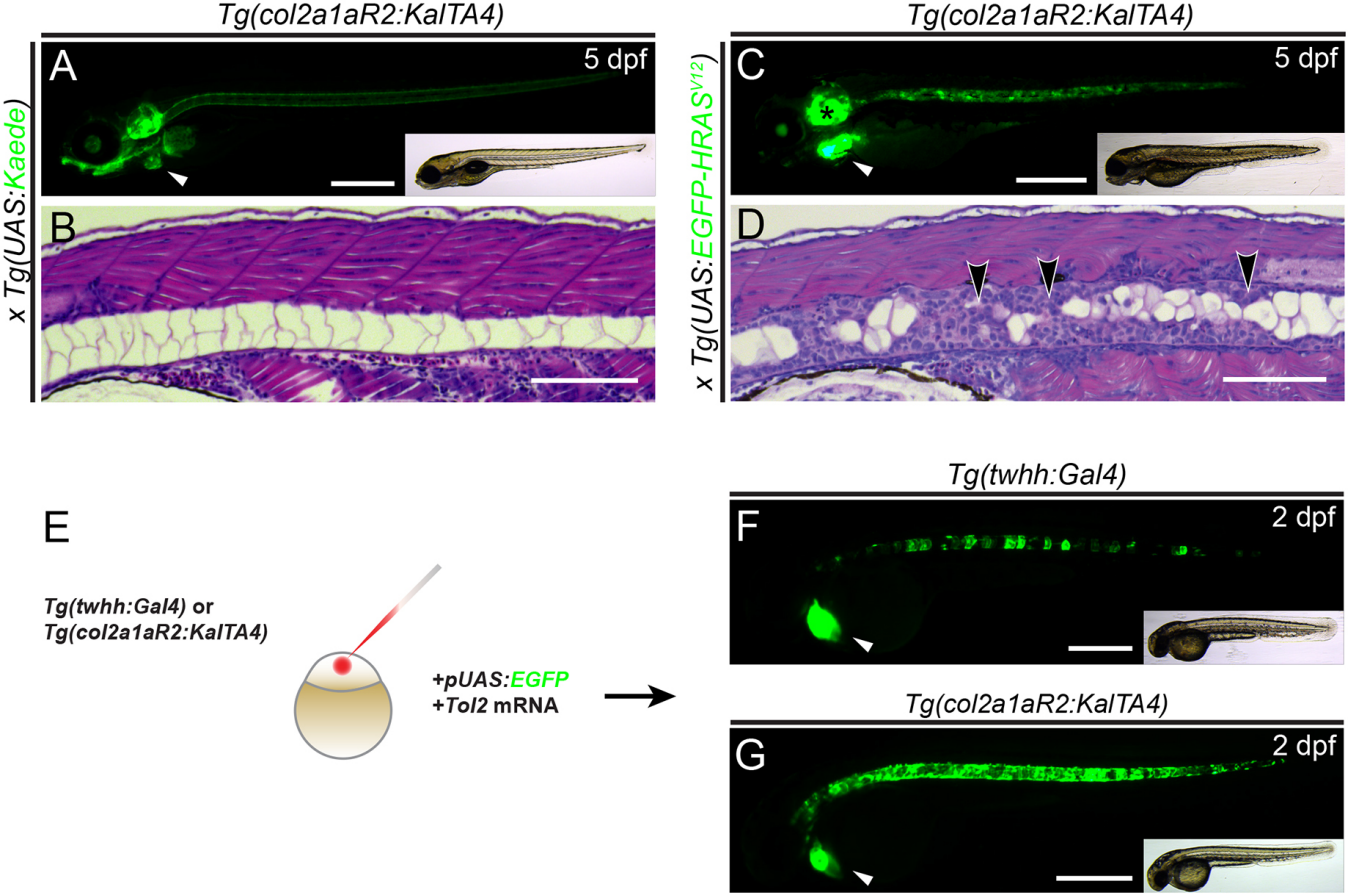
***Tg(col2a1aR2:KalTA4)* enables stable and transient oncogene expression in the developing notochord.** (A,B) Lateral view (A) and transverse histology section (stained with H&E) (B) of 5 dpf zebrafish embryos transgenic for the transgene *col2a1aR2:KalTA4* and crossed with stable *UAS:Kaede* (visible as green fluorescence in A). Note expression in the notochord, craniofacial cartilage, otic vesicle and pectoral fins; the prominent green heart (white arrowhead, A) indicates the *myl7:EGFP* transgenesis marker associated with *col2a1aR2:KalTA4*. The developing notochord shows that the large vacuolated cells take up the vast majority of the notochord volume and are rimmed by a thin layer of sheath cells (B). (C,D) Expression of stable *UAS:EGFP-HRASV12* (detectable by green fluorescence of the fusion protein, C) by *col2a1aR2:KalTA4* causes invasive and widespread notochord hyperplasia (black arrowheads, D) and overgrowth of other cartilage tissue (i.e. otic vesicle, black asterisk in C); the white arrowhead indicates the *myl7:EGFP* transgenesis marker (A). (E-G) *col2a1aR2* provides a potent driver for transient notochord expression. (E) Injection of *UAS:EGFP* into the one-cell-stage embryos with either *twhh:Gal4* (F) or *col2a1aR2:KalTA4* (G) to visualize the notochord mosaicism resulting from random integration of *UAS:EGFP* by Tol2 transposase. While injections into *twhh:Gal4* result in highly patchy EGFP expression (F, green fluorescence; *n*=33/56), *col2a1aR2:KalTA4* more consistently drives EGFP expression throughout the notochord (G, green fluorescence; *n*=25/47); *n* indicates representative EGFP-expressing embryos in an injected representative clutch. Scale bars: 500 μm in A,C,F,G; 200 μm in B,D. See also Fig. S1.

We first sought to functionally assess chordoma-associated genes that could initiate notochord hyperplasia in zebrafish embryos as proxy for the proposed developmental origin of chordoma (Chen et al., 2009; Choi et al., 2008; Heaton and Turner, 1985; Nibu et al., 2013; Romeo and Hogendoorn, 2006). Although our previous chordoma modeling used *twhh:Gal4* as transgene driver, based on the *twhh* promoter region with promiscuous transcriptional activity in notochord cells (Burger et al., 2014; Du and Dienhart, 2001), we aimed for a stronger driver that would also enable injection-based oncogene testing. We generated the *col2a1aR2:KalTA4* transgene that uses the *R2* fragment of the *col2a1a* regulatory region (Dale and Topczewski, 2011) to express the optimized Gal4-based transactivator KalTA4 (Distel et al., 2009) (Fig. 1A,B). When incrossed with stable *UAS* reporters, *col2a1aR2:KalTA4* drove notochord-restricted reporter expression detectable from five- to seven-somite stages (∼12 hpf) covering the entire notochord including sheath cells; expression remained strong throughout development (Fig. 1A,B). Of note, *col2a1aR2*:*KalTA4* activity does not remain restricted to the notochord: at later developmental stages, starting from 2.5 to 3 dpf, *col2a1aR2*:*KalTA4* expression broadly initiated in emerging cartilage lineages, including the otic vesicle, jaw and pectoral fin (Fig. S1A-C); this observation is in agreement with the initial description of *R2* activity (Dale and Topczewski, 2011).

When we crossed *col2a1aR2:KalTA4* with stable *UAS:EGFP-HRAS^V12^*, double-transgenic embryos displayed overproliferation of notochord sheath cells within 2dpf, effectively compressing the inner vacuoles and excluding them altogether in parts of the notochord (Fig. 1C,D). This phenotype was similar to, if not stronger than, the *twhh:Gal4;UAS:EGFP-HRAS^V12^* combination used previously for chordoma modeling in zebrafish (Burger et al., 2014). In addition to the uncontrolled proliferation of notochord cells, starting from 4dpf, *col2a1aR2:KalTA4;UAS:EGFP-HRAS^V12^* embryos developed enlarged otic vesicles and deformed jaw cartilage (Fig. 1C), consistent with the overall expression pattern of the *col2a1aR2:KalTA4* driver (Fig. 1A,C, Fig. S1). These data establish *col2a1aR2:KalTA4* as a transgenic tool to drive *UAS*-based candidate genes in the emerging notochord from early somitogenesis stages (Fig. 1E-G).

### Brachyury overexpression is insufficient to initiate notochord hyperplasia

**Fig. 2.**
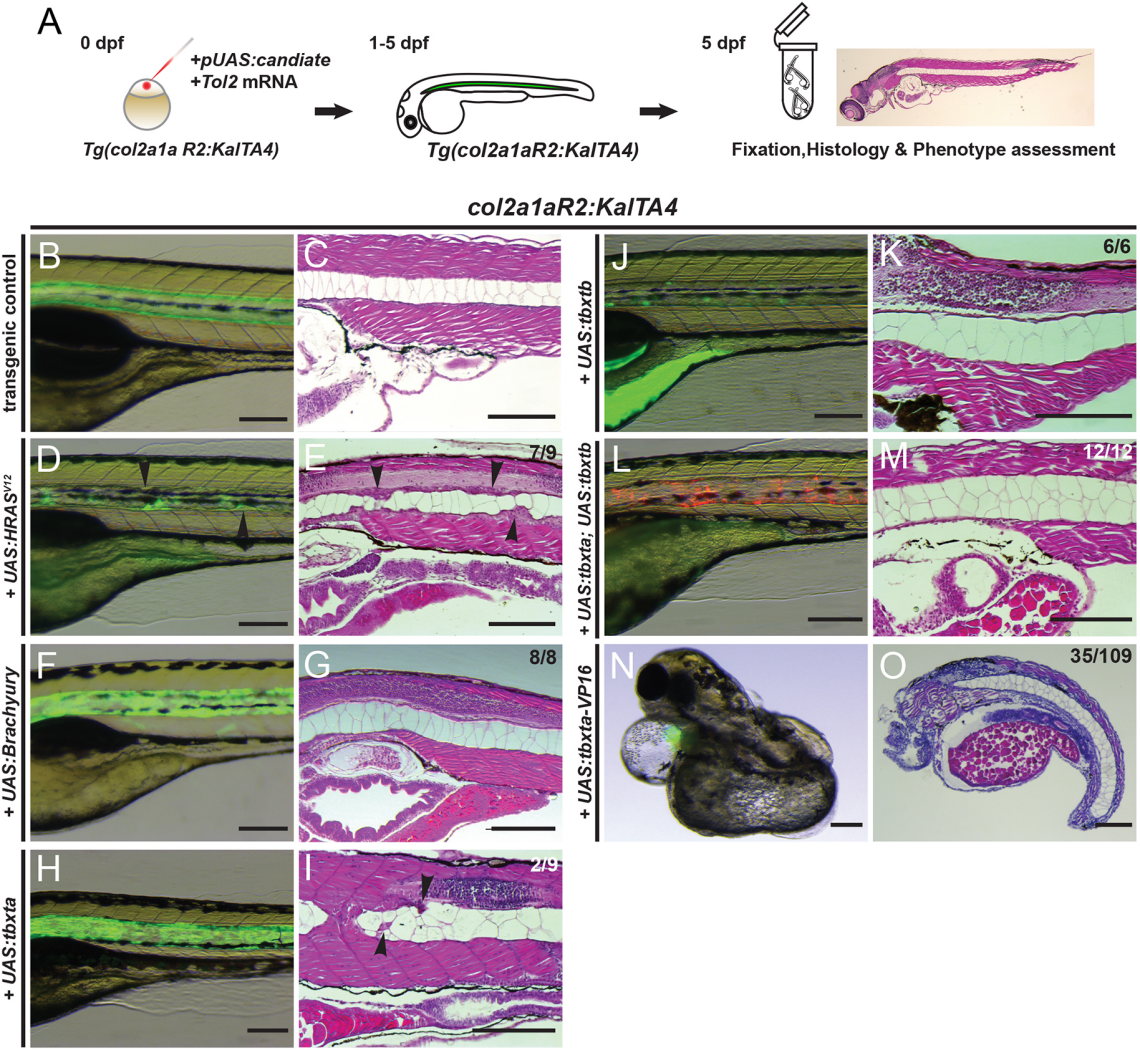
**Overexpression of brachyury genes in the zebrafish notochord is insufficient to initiate chordoma.** (A) Workflow of injection-based notochord hyperplasia assessment: at the one-cell stage, *Tg(col2a1aR2:KalTA4)* embryos are injected with *Tol2* transposase mRNA and a plasmid containing a fluorescently labeled candidate gene under *UAS* control; injected embryos are raised up to 5 dpf and candidate gene expression is monitored through notochord fluorescence. Embryos with consistent reporter expression are fixed, sectioned and stained with H&E to assess the notochord phenotype using light microscopy. (B-O) Close-up lateral view of embryo notochords at 5 dpf, for brightfield and fluorescence (left column) and H&E histology (right column; different embryos per condition); numbers indicate observed versus total from an individual representative experiment. (B,C) The *col2a1aR2:KalTA4* control reference at 5 dpf, expressing *UAS:Kaede* to fluorescently label the notochord. (D,E) Transient injection of *UAS:EGFP-HRAS^V12^* causes localized notochord hyperplasia (arrowheads). (F-I) Forced expression of human *TBXT* (brachyury) (F,G) or of the zebrafish gene *tbxta* (H,I) does not affect notochord development or cell proliferation. Minor lesions caused by collapsed vacuolated cells developed in a few *UAS:tbxta*-expressing notochords (arrowheads, I). (J,K) Overexpression of the second zebrafish brachyury gene *tbxtb* does not affect notochord development or proliferation. (L,M) Combined overexpression of both zebrafish brachyury genes *tbxta* and *tbxtb* has no effect on proliferation and the notochord develops normally. (N,O) Notochord-driven expression of *tbxta-VP16*, encoding a dominant-active transcriptional activator, leads to non-autonomous defects in the trunk with a shortened and/or severely curled trunk (32% of analyzed embryos), whereas the notochord forms normally. Scale bars: 200 μm. See also Fig. S2.

We next assessed whether we can perform candidate gene tests by transforming notochord cells with transient injections into *col2a1aR2:KalTA4* embryos (Fig. 2A). Injection of Tol2 transposon-based constructs into zebrafish results in mosaic transgene expression owing to the random nature of Tol2 transposon integrations (Kawakami, 2004; Koga et al., 1996; Kwan et al., 2007). Although not covering all developing cells, injected Tol2 transgenes result in clonal heterogeneity of transgene copies throughout the embryos, providing a proxy for the different gene dosage and genetic mosaicism also found in cancer (Ceol et al., 2011; Kaufman et al., 2016). Closely recapitulating the phenotype of stable *HRAS^V12^* overexpression (Fig. 1C,D) and corresponding to the mosaic pattern of the transient *HRAS^V12^* expression resulting from *UAS* construct injection (Fig. 1E-G), transient *HRAS^V12^* overexpression consistently caused scattered localized tumorigenic lesions along the notochord (Fig. 2B-E).

The pathological detection of TBXT protein and the frequently found copy number gains of the *TBXT* locus, as hallmarks of human chordoma, have been hypothesized to represent tumor-causing insults. We therefore used *col2a1aR2:KalTA4* to test the potential of increased brachyury expression to transform the developing zebrafish notochord. We cloned *UAS* vectors harboring full-length human *TBXT* and its main zebrafish ortholog *tbxta* coupled in *cis* with *UAS:EGFP* to avoid possible inactivating tagging of brachyury proteins (*UAS:TBXT,UAS:EGFP* and *UAS:tbxta,UAS:EGFP*, respectively). We chose this strategy as direct tagging of brachyury open reading frames (ORFs) with fluorescent proteins led to inconsistent transgene expression, possibly indicating aberrant protein folding or activity (Fig. S2A-F). In contrast, upon injection of untagged *UAS:EGFP*-coupled *UAS-brachyury* or *UAS-tbxta* into *col2a1aR2:KalTA4* embryos, we observed reproducible EGFP fluorescence signal throughout the notochord; moreover, all embryos expressing either human *TBXT* or zebrafish *tbxta* developed normal-appearing notochords within the observed 5dpf (Fig. 2F-I). Consistent with the injection-based results, embryos carrying a stable *UAS:tbxta* transgene and *col2a1aR2:KalTA4* also developed normal notochords (Fig. S2G,H). Contrary to humans and mice that harbor a single copy of the gene, zebrafish harbor two paralogs, *tbxta* and *tbxtb* (Martin and Kimelman, 2008). Nonetheless, the overexpression of *UAS:tbxtb* (coupled *in cis* with *UAS:mCherry* to avoid tagging) alone did not affect the integrity of the notochord (Fig. 2J,K), nor did the combined overexpression of *UAS:tbxta* and *UAS:tbxtb* within the observed 5 dpf (Fig. 2L,M).

To augment the activity of zebrafish *tbxta* as transcriptional activator, we expressed a Tbxta-VP16 fusion protein in which native Tbxta is fused with the strong VP16 transactivation domain; Tbxta-VP16 strongly augments the transcriptional activity of Tbxta in zebrafish embryos (Bruce et al., 2003). The *col2a1aR2:KalTA4*-driven notochord expression of transiently injected *UAS:tbxta-VP16* reproducibly resulted in embryos with severe body curvature, a shortened body axis and cardiac edema, yet analyzed notochords remained structurally intact and devoid of any hyperplasia (Fig. 2N,O).

From these results, within the timeframe of our observations, we conclude that increased expression of *ntl/tbxta/tbxtb*, even of a transcriptionally hyperactive form, is insufficient per se to transform developing zebrafish notochord cells into a hyperplastic state.

### Overexpression of RTK genes found to be activated in human chordoma is sufficient to initiate notochord hyperplasia

RTKs and their branched downstream pathways are key players in development (Casaletto and McClatchey, 2012), and activation of RTKs and their downstream Ras-dependent cascades can transform a variety of cell types into tumors (Regad, 2015; Sangwan and Park, 2006). Although activated Ras mimics upstream RTK activation, as used in our original chordoma model (Burger et al., 2014), Ras mutations are seemingly rare in chordoma (Choy et al., 2014; Tauziède-Espariat et al., 2016). By contrast, an increasing number of studies have reported copy-number alterations, increased phosphorylation or misexpression of individual RTKs in chordoma, most prominently of *EGFR*, *KDR*, *PDGFRA*, *KIT* and of different FGFR genes (Akhavan-Sigari et al., 2014a,b,c; de Castro et al., 2013; Dewaele et al., 2011; Fischer et al., 2015; Launay et al., 2011; Miettinen et al., 2012; Tamborini et al., 2006).

**Fig. 3.**
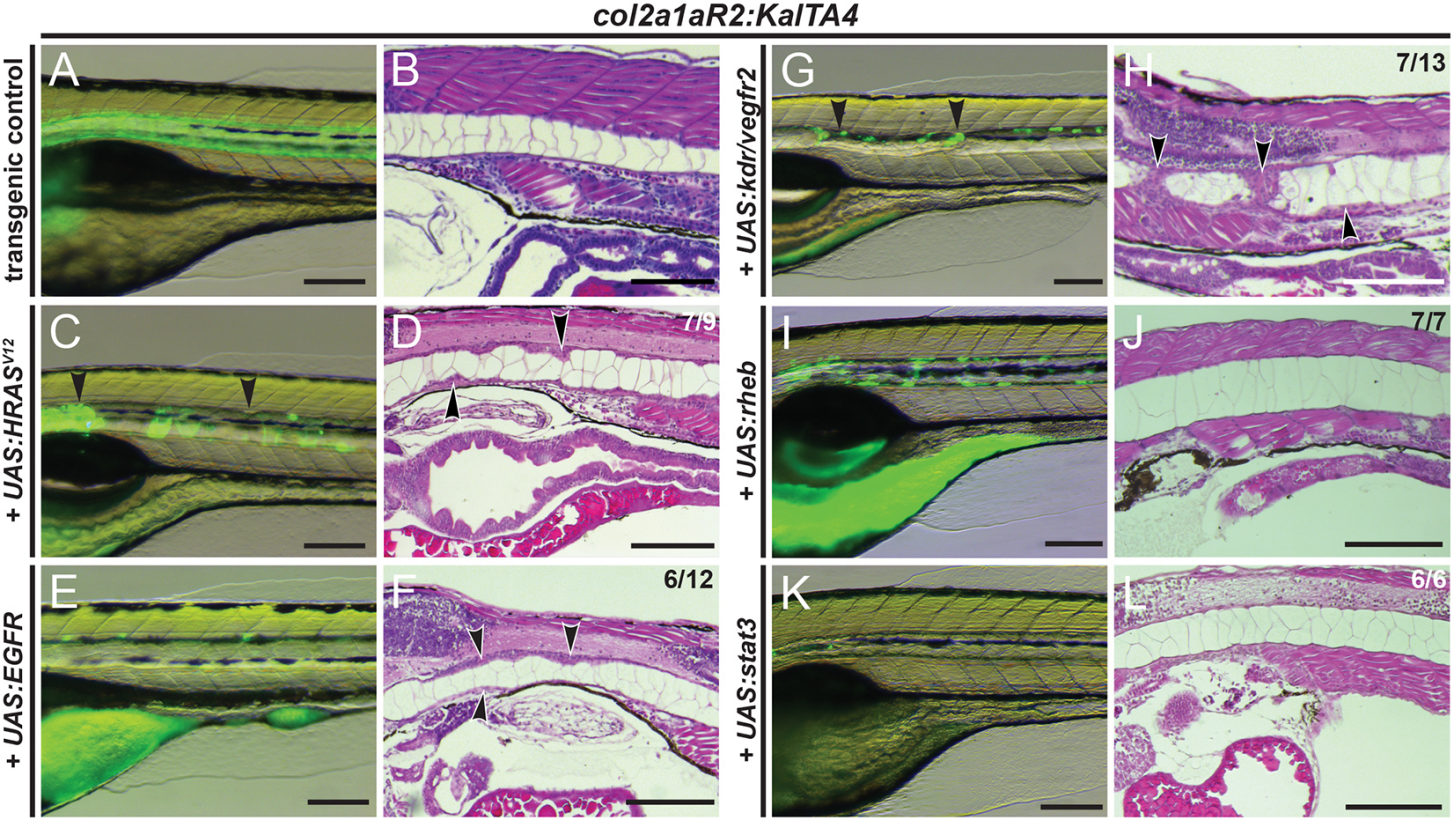
**Transient overexpression of RTK genes drives notochord hyperplasia.** (A-L) Close-up lateral views of embryo notochords at 5 dpf, with brightfield/fluorescence (left panels) and H&E histology (right panels; different embryos per condition); numbers in D,F,H and J indicate embryos with observed lesions versus phenotypically normal embryos observed in an individual representative experiment, and numbers in L indicate wild-type-looking embryos (*n*=3 experiments). (A,B) The *col2a1aR2:KalTA4* control reference at 5 dpf, expressing *UAS:Kaede* to fluorescently label the notochord. (C,D) Transient injection of *UAS:EGFP-HRAS^V12^* causes localized hyperplasia (arrowheads, C,D) in the notochord. (E,F) Overexpression of human *EGFR* consistently causes local hyperplasia in the developing notochord (arrowheads, F). (G,H) Overexpression of zebrafish *kdr* (encoding the *VEGFR2* ortholog) causes strong hyperplasia (arrowheads in G,H, compare with D,F). (I,J) Zebrafish *rheb* overexpression leads to enlarged vacuoles and no detectable hyperplasia. (K,L) Zebrafish *stat3* overexpression leads to no detectable hyperplasia and allows for normal notochord development within 5 dpf. Scale bars: 200 μm. See also Fig. S3.

To test the potential of misexpressed individual RTKs for driving hyperplastic notochord transformation, we transiently injected *col2a1aR2:KalTA4* embryos with *UAS* constructs harboring the full-length ORFs of candidate RTKs and additional chordoma-implicated candidate genes as reference. Mosaic expression of *UAS* constructs for zebrafish *c-kit*, *pdgfra*, *fgfr3* and *fgfr4* all resulted in high mortality rates during somitogenesis (>80% in the case of *fgfr3* and *fgfr4*): in injected embryos, we frequently observed severe body axis perturbations and dorso-ventral patterning defects (Fig. S3). As *col2a1aR2:KalTA4* drives these transgenes selectively in the notochord early on, these phenotypes suggest severe non-autonomous effects upon notochord-focused expression of *c-kit*, *pdgfra*, *fgfr3* and *fgfr4*, precluding further analysis using our approach. By contrast, and comparable to the injection of *UAS-HRAS^V12^* (Fig. 3A-D), mosaic notochord-driven expression of both *UAS:EGFR* and *UAS*:*kdr*, potently triggered sheath-cell hyperplasia between 2-5dpf (Fig. 3E-H). Both EGFR- and Kdr-expressing notochords displayed localized hyperplasia along their entire length (Fig. 3F,H): we repeatedly observed clusters of overgrowing cell patches in the center of the notochord, which compress the vacuolated inner notochord cells, as also observed in *HRAS^V12^* expression (Figs 2E and 3D).

Besides TBXT and RTKs, several other signaling factors have been found to be activated or misexpressed in chordoma (Frezza et al., 2019; Wasserman et al., 2018). Phosphorylation of mTOR, a key downstream mediator of RTK/Ras signaling in the mTORC1 and mTORC2 complexes that control ribosome biogenesis and protein synthesis (Manning and Toker, 2017), has been repeatedly found in chordoma (Presneau et al., 2009). The small GTPase Rheb activates mTORC1 and can act upon overexpression as a proto-oncogene (Armijo et al., 2016; Garami et al., 2003; Nardella et al., 2008; Yang et al., 2017). Nevertheless, *col2a1a:KalTA4* embryos injected with *UAS:rheb* did not develop any notochord hyperplasia within the observed 5dpf (Fig. 3I,J). We further tested the tumorigenic potential of *STAT3* involved in JAK/STAT signaling, as positive staining for STAT3 and phospho-STAT3 have been repeatedly reported in human chordoma (Dobashi et al., 2007; Tauziède-Espariat et al., 2016; Yang et al., 2010). Nonetheless, *col2a1aR2:KalTA4* embryos expressing *UAS:stat3* also developed normally without any signs of notochord hyperplasia during the first 5dpf (Fig. 3K,L). Taken together, our results suggest that of the chordoma-implicated genes we successfully tested in our assay, only overexpression or misexpression of the chordoma-implicated EGFR and KDR RTKs is sufficient to trigger the onset of notochord hyperplasia.

**Fig. 4.**
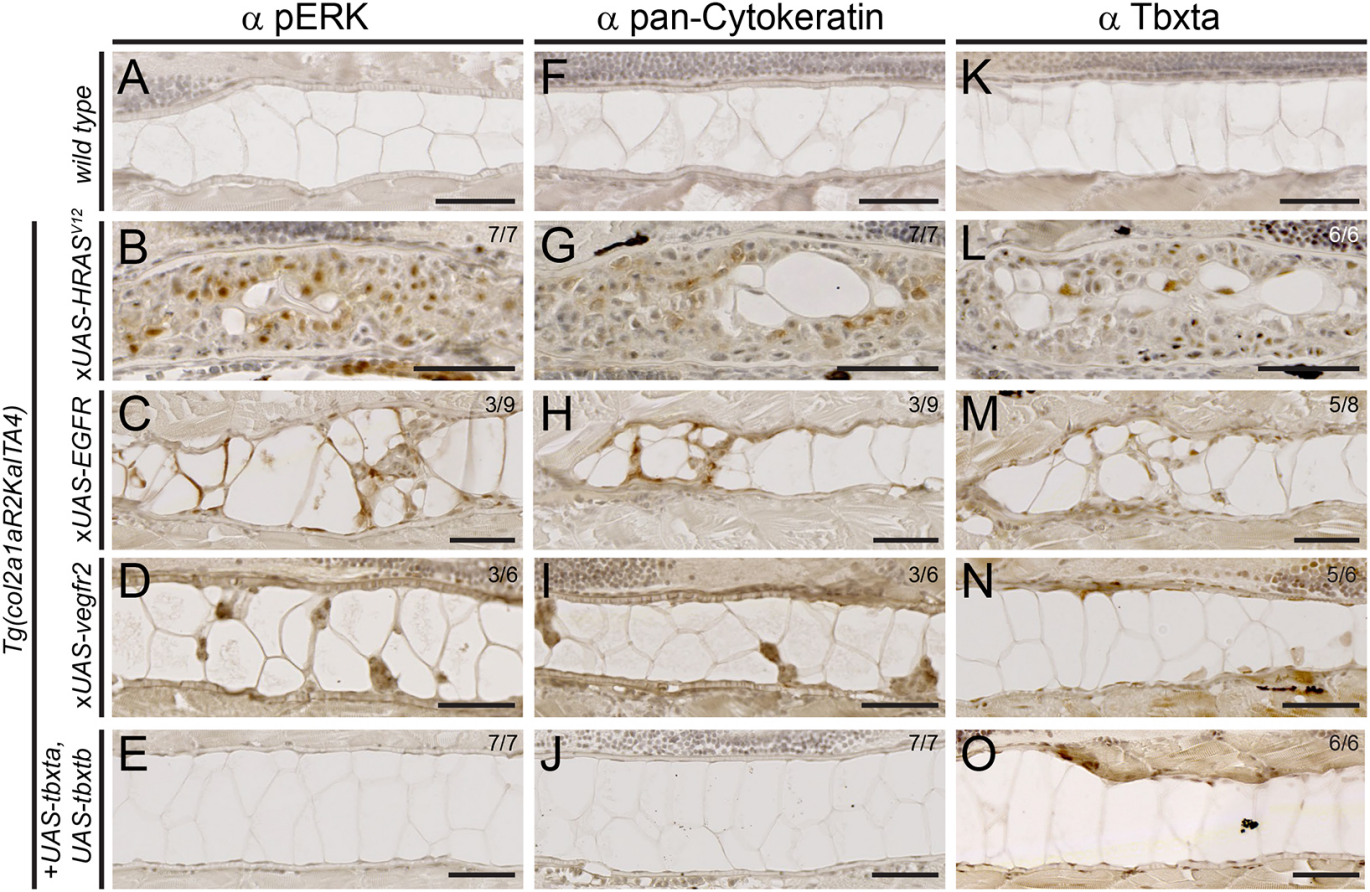
**Expression of chordoma markers in RTK-transformed zebrafish notochords.** (A-O) Immunohistochemistry on sagittal sections through the notochord of 5 dpf zebrafish embryos of the indicated genotypes, expressing either stable or mosaic transgenes. (A-E) MAPK pathway activation through *HRAS^V12^*, *EGFR* and *kdr* overexpression results in nuclear pERK staining in the notochord, whereas controls and *tbxta*,*tbxtb-*injected embryos are negative for pERK staining. (F-J) *HRAS^V12^*, *EGFR* and *kdr* overexpression results in staining for pan-Cytokeratin, whereas *tbxta*,*tbxtb-*injected embryos are negative for pan-Cytokeratin staining akin to wild-type controls. (K-O) Whereas control notochords show faint to no Tbxta signal, owing to low cell density of the sheath layer (K), *HRAS^V12^*-overexpressing notochords (L) as well as *EGFR-*overexpressing (M) and *kdr*-overexpressing (N) notochords show prominent nuclear Tbxta staining. The *tbxta*,*tbxtb*-overexpressing notochords also stain positive (O), confirming transgenic *tbxta* expression. Scale bars: 50 µm. See also Fig. S4.

We confirmed that *EGFR-* and *kdr*-misexpressing cells have activated MAPK signaling, as revealed by probing for the downstream effector pERK: compared with 5 dpf wild-type notochords that are devoid of pERK (Fig. 4A), *HRAS^V12^*, *EGFR* and *kdr* misexpression resulted in pERK-positive notochord cells, including prominent staining in cells infiltrating the notochord (Fig. 4B-D). Compared with controls, the *HRAS^V12^*-, *EGFR-* and *kdr-*overexpressing notochords also stained notably stronger for the common chordoma marker pan-Cytokeratin (Fig. 4F-I). We also observed staining for zebrafish protein Tbxta (Fig. 4K-O; see Fig. S4 for human *TBXT* antibody). Consistent with the absence of hyperplastic cells, combined overexpression of the zebrafish *tbxta* and *tbxtb* ORFs did not change the staining for any of these markers (except for the misexpressed Tbxta itself, accumulating in nuclei) compared with wild type (Fig. 4E,J,O). These observations suggest that commonly used diagnostic markers for human chordoma stain positive in hyperplastic zebrafish notochords with activated RTK signaling.

Taken together, in zebrafish, increased levels of EGFR and Kdr are sufficient to induce hyperplasia of developing notochord sheath cells by triggering RTK signaling, akin to constitutively active *HRAS^V12^*. By contrast, notochord-driven expression of effectors of mTOR or JAK/STAT signaling, or of different versions of brachyury (Fig. 2F-O), did not trigger overproliferation with early onset. These observations raise the possibility that aberrant activation of RTK signaling is a key process to trigger notochord cell hyperplasia, whereas other chordoma-implicated mechanisms could then potentially contribute after the initial tumor onset.

### Ras-mediated notochord transformation in zebrafish recapitulates abnormalities found in human chordoma

**Fig. 5.**
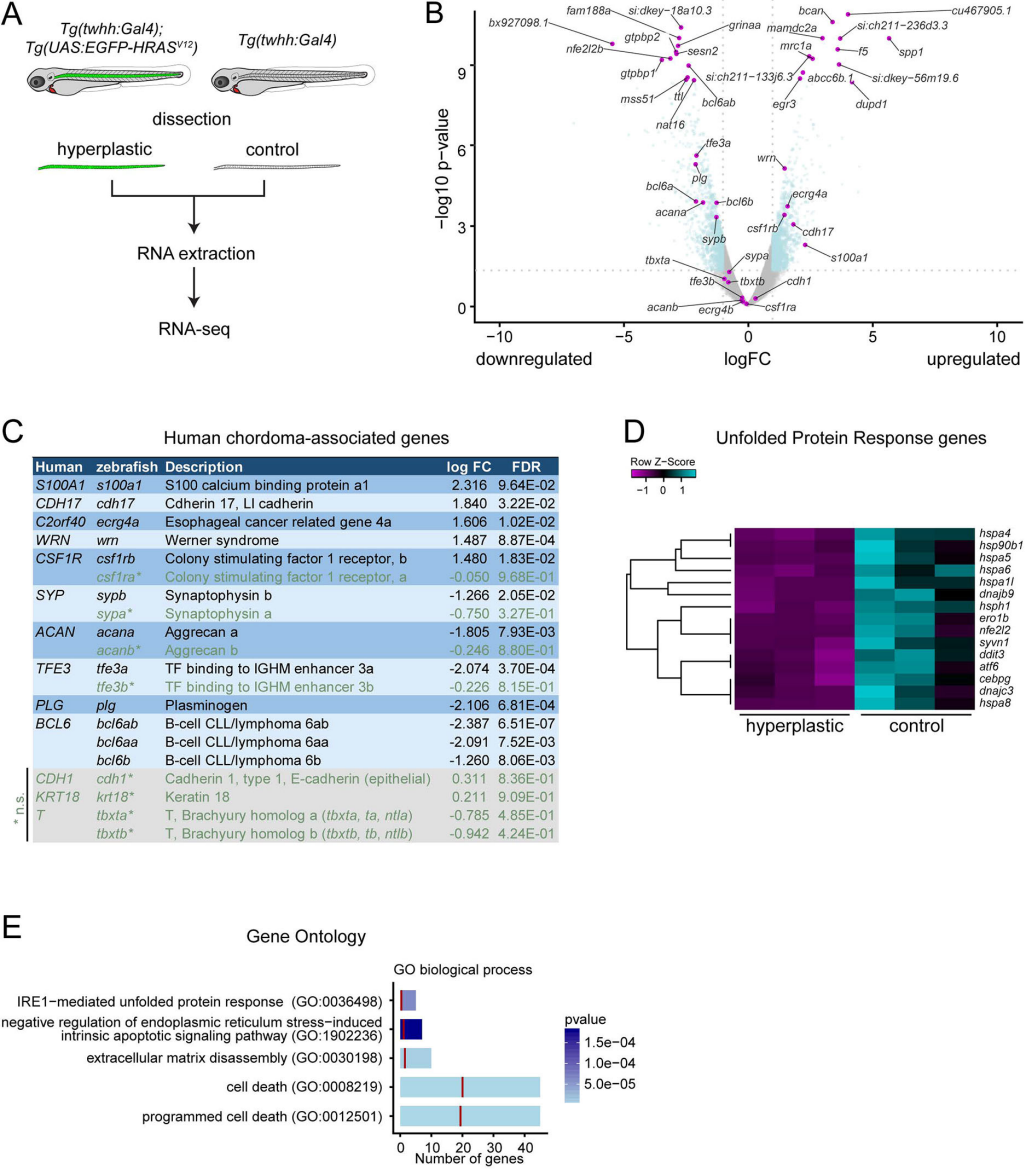
**HRAS^V12^-induced zebrafish chordomas have a deregulated UPR and suppress apoptosis.** (A) Workflow of control and transformed notochord isolation. Notochords were dissected from *twhh:Gal4* (wild-type morphology and transgenic base line) and *twhh:Gal4;UAS:EGFP-HRAS^V12^*-expressing larvae at 8 dpf; see Materials and Methods for details. (B) Volcano plot depicting overall distribution of deregulated genes: gray, genes for which *P*<0.05; blue, genes with significant deregulation between control versus transformed notochords. Note that the zebrafish brachyury genes *tbxta* and *tbxtb* are unchanged but expressed. (C) Expression of human chordoma-associated genes in *HRAS^V12^*-transformed zebrafish notochords; see text for details. Zebrafish orthologs of human chordoma genes are shown with all their orthologs: orthologs with changes considered to be not significant (n.s., green) are marked with asterisks, genes with no significantly altered ortholog are shown in gray boxes at the bottom. (D) Significantly deregulated UPR genes as per IPA pathway analysis of hyperplastic versus control zebrafish notochords. (E) Gene ontology (GO) enrichment in hyperplastic zebrafish notochords also highlights deregulation of the UPR, ER stress, ECM dynamics and cell death. The vertical red bars represent the expected number of genes per GO term under random selection based on the reference list, *Homo sapiens* REFLIST (21,042 genes in total). See also Fig. S5.

To gain more insight into the initial phase of notochord hyperplasia triggered by Ras-mediated notochord transformation in zebrafish, and to assess whether genes associated with human chordoma become deregulated, we performed RNA sequencing (RNA-seq) analysis of dissected wild-type notochords versus *HRAS^V12^*-transformed notochords (Fig. 5A).

A total of 591 genes were found to be significantly deregulated in hyperplastic notochords compared with wild type (228 upregulated, 363 downregulated; minimum log fold change ≥1, corrected FDR ≤0.05) (Fig. 5B, Table S1). Notably, in the analyzed zebrafish control notochords, we detected modest but significant expression of both *tbxta* and *tbxtb*, as confirmed by reverse transcriptase PCR (RT-PCR) (Fig. 5B, Fig. S5A-C). Neither *tbxta* nor *tbxtb* significantly changed upon *HRAS^V12^* transformation (Fig. S5A-C, Table S1). These data indicate that, in the developing notochord, the expression of the zebrafish *tbxta* and *tbxtb* genes persists beyond the initial embryonic stages for considerably longer than commonly assumed based on mRNA *in situ* hybridization (San et al., 2016; Schulte-Merker et al., 1992).

Among the significantly deregulated genes, we found genes that have previously been implicated in being aberrantly expressed in human chordoma. For example, loss of the *BCL6* locus has been reported as a possible frequent event in chordoma (Rinner et al., 2013). Consistent with this notion, all zebrafish Bcl6 family genes, in particular *bcl6ab*, were significantly downregulated in our *HRAS^V12^*-transformed zebrafish notochords (Fig. 5B,C, Table S1). Furthermore, *S100A1* is a potent diagnostic marker in clinical chordoma cases: zebrafish *s100a1* was also the most significantly upregulated *s100* gene in our hyperplastic zebrafish notochords, albeit under a slightly less stringent FDR threshold (logFC=2.32, *P*=0.006, FDR=0.096) (Fig. 5B,C, Table S1). We conclude that, despite species differences, the induction of notochord hyperplasia in zebrafish using activation of the RTK/Ras cascade deregulates similar genes to those observed in human chordoma.

We also performed downstream pathway analysis of the deregulated genes using both Ingenuity Pathway Analysis (IPA) and Gene Set Enrichment Analysis. Both of these analyses revealed a significant downregulation of genes associated with ER stress and with the UPR (Fig. 5D,E, Fig. S5D-G). Given the established roles of the UPR in notochord development and differentiation towards mineralized bone (Ishikawa et al., 2013, 2017a; Tanegashima et al., 2009), these data suggest that an early response of the notochord to RTK/Ras transformation is a suppression of this normal developmental program. Supporting this notion, other affected pathways included processes involved in bone and cartilage biology, including the deregulation of markers of bone differentiation such as *spp1*, *anxa5* and *ihha* (Fig. S5E,G). In addition, we noted a significant deregulation of genes associated with ECM remodeling (Fig. 5E), a process associated with notochord sheath cells for building up a thick ECM around the forming notochord before the onset of segmented ossification (Dale and Topczewski, 2011; Fleming et al., 2004; Gray et al., 2014; Grotmol et al., 2003). Taken together, the transcriptome of notochord cells transformed by activated RTK/Ras signaling shows hallmarks of suppressed notochord differentiation.

**Fig. 6.**
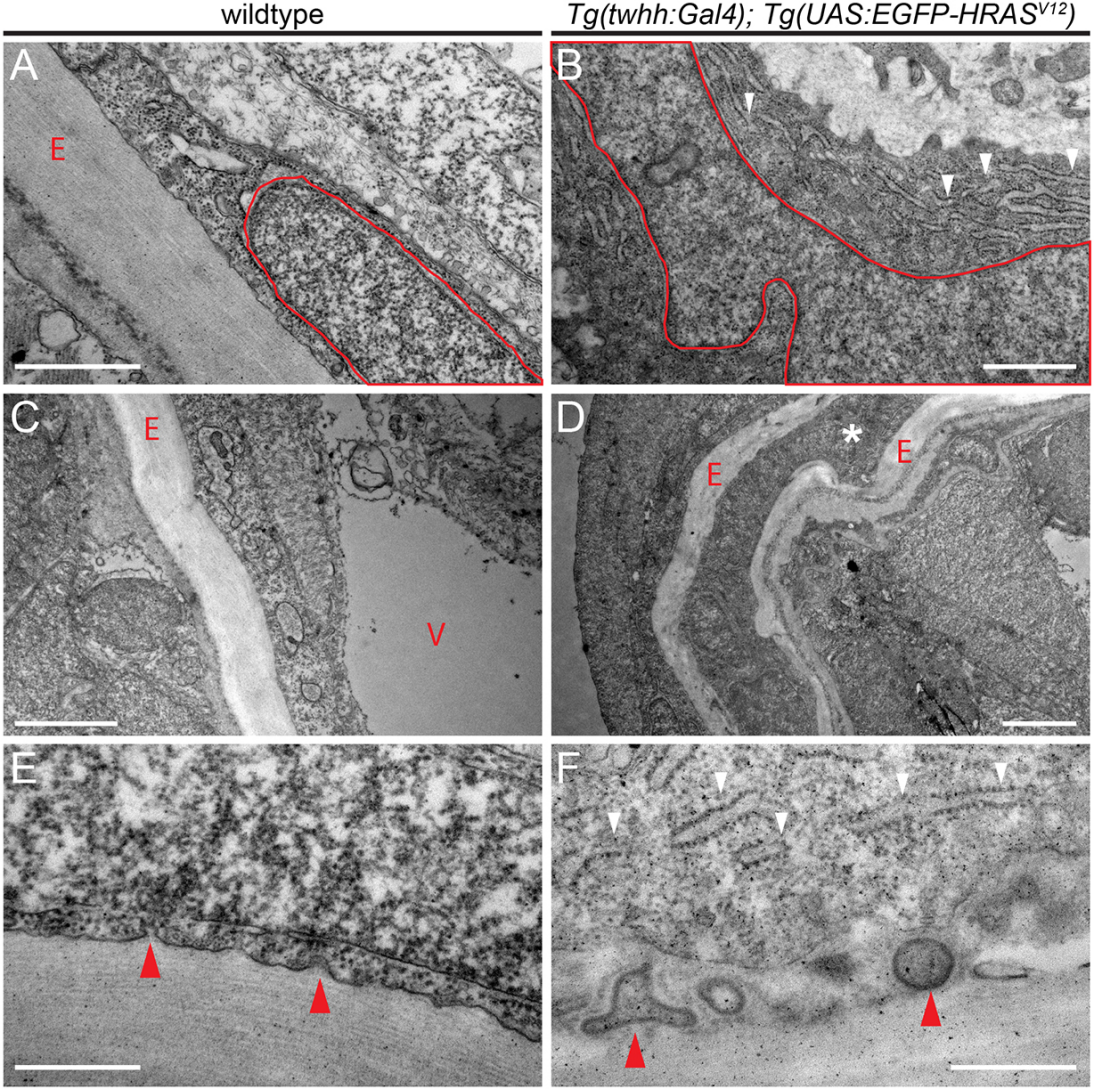
**Aberrant ECM and ER accumulation in *HRAS^V12^*-induced zebrafish chordomas.** (A-F) Transverse sections through wild-type (A,C,E) and *HRAS^V12^*-transformed (B,D,F) zebrafish notochord at 8 dpf imaged using TEM. (A,B) Wild-type cells have a pill-shaped, regular nucleus (red outline, A) and form regular ECM layers (red letter ‘E’) secreted by the sheath cells at the outside of the notochord. Nuclei in transformed cells expand and develop lobed and distorted nuclear shapes (red outline, B); transformed cells further accumulate extensive ER lumen (white arrowheads, B). (C,D) In wild-type notochords, vacuolated cells (red letter ‘V’) take up the majority of the space inside the ECM-lined notochord (red letter ‘E’) to provide mechanical stability; transformed notochords become filled with non-vacuolated cells and secrete aberrant amounts of ECM that leads in extreme cases to entombed cells trapped in ECM layers (white asterisk, D). (E,F) Membrane details of wild-type versus transformed notochord sheath cells. Wild-type notochords show budding of vesicles that transport collagen for stereotypically layered ECM build up (red arrowheads); in transformed notochords, the secretion process appears to be overactive (ER accumulation shown by white arrowheads, F) and results in membrane inclusions within the ECM (red arrowheads). Scale bars: 1 μm (A,B); 2 μm (C,D); 0.5 μm (E,F). See also Fig. S6.

To observe the cellular consequences of early notochord transformation, as indicated by our transcriptome analysis, we performed electron microscopy (EM). Transverse sections analyzed by EM again documented sheath cells in the center of the notochord, resulting in compression of the inner vacuoles (Fig. S6A,B). Strikingly, and in contrast to the small elliptical nuclei that occur in wild-type sheath cells, transformed notochord cells developed highly enlarged nuclei with irregular and lobulated shapes (Fig. 6A,B), as described previously for human chordoma (Kolb et al., 2014). Notably, transformed notochord cells showed an increase in ER membranes throughout the analyzed transformed sheath cells (Fig. 6A,B), indicating highly active secretion that builds up unorganized ECM layers (Fig. 6C,D, Fig. S6). Zebrafish chordomas generated using our approach do not metastasize (Burger et al., 2014; this study) but remain confined to the notochord. Ultimately, the ECM could be responsible for the absence of detectable outgrowth from the notochord in our zebrafish model: we observed several instances of individual sheath cells that had seemingly detached from the notochord and had become completely entombed within the secreted ECM (Fig. 6D). We detected a high abundance of secretory vesicles budding with the cell membrane in wild-type notochords, whereas transformed notochord cells featured amorphic cell boundaries to the ECM with trapped membrane fragments embedded in the collagen matrix (Fig. 6E,F).

Taken together, these observations suggest that RTK/Ras cascade-triggered sheath-cell hyperplasia has the hallmarks of deregulated ER stress and UPR, key processes involved in the control of the progenitor versus differentiation program in the notochord. Oncogenic transformation by aberrantly activated RTK signaling could therefore drive the maintenance of an incompletely differentiated, developmental state in transformed notochord cells. By contrast, Brachyury expression is ongoing in the zebrafish notochord both in wild-type and in transformed conditions, indicating that maintained Brachyury expression reflects notochord identity of the transformed cells.

## DISCUSSION

Although assumed to represent a notochord-derived tumor of embryonic remnant cells, the exact ontogeny and hyperplasia-inducing events leading to chordoma remain unclear. To our knowledge, activating Ras mutations have so far not been reported from chordoma samples, and a comprehensive comparison of the zebrafish-based chordoma transcriptome with human chordoma is hindered by the lack of readily accessible transcriptome data for this rare tumor. Nevertheless, several RTK genes have been repeatedly found to be aberrantly activated or amplified in human chordoma samples. Harnessing our zebrafish-based notochord readouts as proxy for chordoma onset, we evaluated the potential of several chordoma-implicated candidate genes for their capacity to transform native notochord cells *in vivo*, both with injection and verified with stable transgenic insertions. In our assays, the RTKs EGFR and KDR, both abundantly found as oncogenes in various other cancer types, robustly triggered notochord hyperplasia. By contrast, overexpression of various forms of brachyury, including as hyperactive VP16 fusion protein, caused no apparent notochord transformation in our observed timeframe. These results provide the first direct functional testing of the chordoma-inducing potential of implicated oncogenes in native notochord cells.

We performed overexpression of brachyury in the developing notochord by different means to avoid use of functionally impaired fusion proteins (Fig. S2A-E). Misexpression of human *TBXT*, as well as individual or combined expression of zebrafish *tbxta* and *tbxtb*, consistently failed to induce notable notochord hyperplasia (Fig. 2F-M). Of note, misexpression of these genes did occasionally cause aberrant notochord architecture, with collapsed sheath cells reminiscent of recent reports of structural lesions in notochord morphology (Fig. 2H,I) (Garcia et al., 2017; Lopez-Baez et al., 2018). Most compelling is the inability of *tbxta-VP16* to transform the notochord: *tbxta-VP16* encodes a previously validated (Bruce et al., 2003) highly transcriptionally active fusion protein through the viral VP16 transactivation domain (Fig. 2N,O). These results provide the first *in vivo* evidence that, within the first 5 dpf in zebrafish, misexpression of Brachyury in developing notochord cells is insufficient to elicit a hyperplastic response.

Besides Brachyury, pathological detection of several other factors has been recurrently reported in chordoma cases. Ras/PI3K/AKT pathway activators, mainly RTKs including EGFR and KDR, are frequently found to be activated or copy number-amplified in chordoma patients (Akhavan-Sigari et al., 2014a,b,c; de Castro et al., 2013; Fischer et al., 2015; Miettinen et al., 2012; Presneau et al., 2009; Tamborini et al., 2006). Furthermore, promising advances in experimental chordoma treatment have employed RTK inhibitors, specifically compounds targeting EGFR (Asklund et al., 2014; Stacchiotti et al., 2013). Our results from assessing the hyperplasia-inducing potential of individual factors suggest that several RTKs are potent, and possibly redundant, oncogenes when aberrantly activated in notochord cells (Fig. 3E-H). Although RTKs relay crucial signals during embryo development, as underlined by the severe developmental defects caused by notochord-focused misexpression of FGFR, *Kit* and PDGFR genes (Fig. S3A-D), the repeatedly chordoma-associated *EGFR* and *KDR* are, in our assay, individually sufficient to transform developmental notochord cells (Fig. 3E-H). Phosphorylated mTOR, the core component of mTORC1 and mTORC2 downstream of activated RTKs (Manning and Toker, 2017), was found in a number of chordoma cases (Han et al., 2009; Presneau et al., 2009; Tamborini et al., 2010). Nonetheless, activating mTORC1 through misexpression of its direct upstream regulator Rheb is insufficient to trigger hyperplasia (Fig. 3I,J), suggesting that additional events downstream of RTK/Ras activation are required for triggering notochord hyperplasia. STAT3-based signaling has been implicated in several chordoma studies (Fasig et al., 2008; Yang et al., 2009, 2010), yet does not seem to be universally activated in chordoma. In our assay, misexpression of wild-type *stat3* had no transformative affect on the notochord (Fig. 3K,L), suggesting the potential of STAT3 to promote chordoma after the tumor-initiating hits.

Of note, our results with negative hyperplasia outcome do not rule out a chordoma-promoting potential for Brachyury, STAT3 or any other tested factor. Our assay is confined to an early developmental time window, which has the potential to reveal the sufficiency of aggressive notochord-transforming factors. Our results instead suggest that chordoma-initiating events are most-potently mediated by triggering upstream events of RTK signaling; further work is warranted to analyze the synergy and combinatorial action of the individual chordoma lesions found in particular patients or across the analyzed tumors so far. In particular, the striking consistency of *TBXT* expression in chordoma has gathered increasing attention as a tumor-defining and possibly causative characteristic, pointing at the re-activation or maintenance of the embryonic notochord program as the cause (Nelson et al., 2012; Presneau et al., 2011; Szuhai and Hogendoorn, 2012; Yang et al., 2009). Nonetheless, developmental perturbation of *T* during mouse notochord formation suggests a role in maintaining cellular notochord identity, without having any effects on proliferation or other cellular phenotypes upon perturbation (Zhu et al., 2016). Furthermore, the relevance of *TBXT* expression for patient outcome remains unclear.

Two possibilities could account for the prominent expression of brachyury in chordoma. First, in line with previous reports (Tamplin et al., 2011; Zhu et al., 2016), brachyury expression reflects the notochord identity of transformed notochord remnants and maintenance of an early notochord program in chordoma. Several upstream signaling pathways influence brachyury expression during development by acting on *cis*-regulatory elements that remain incompletely charted. Consequently, brachyury expression in transformed notochord cells might reflect the sustained activity of the developmental notochord program. This conclusion is further supported by our observation of persistent *tbxta/tbxtb* expression in zebrafish that did not significantly react to RTK/Ras activation (Fig. 5B, Fig. S5B,C, Table S1), and similar reports of sustained expression in mouse (Zhu et al., 2016) and human (Rodrigues-Pinto et al., 2018).

Alternatively, not acting as transforming agent itself, brachyury expression could feed into the aberrant transcriptional program ongoing in chordoma cells after initial transformation. In chordoma, concomitant elevated brachyury expression could result in additional or combinatorial events that direct RTK-transformed cells into a hyperproliferative state conducive to tumor progression and metastasis formation. Congenital amplification of the gene locus could confer sensitivity to notochord cells for aberrant RTK activation and for subsequently faster tumor formation (Yang et al., 2009). Knockdown of *T* in the developing notochord in mouse has revealed that its function was dispensable for progenitor cell survival, proliferation and EMT (Zhu et al., 2016), and it would be interesting to investigate the effect of brachyury knockdown in a Ras-overexpressing background using a mammalian model system. Expressed beyond physiological levels for a prolonged time, brachyury could nonetheless contribute aberrantly to tumorigenic events. For example, it has been linked to the RTK-based FGF pathway by controlling production of the FGF2 ligand to possibly maintain a positive feedback loop, resulting in a mesenchymal phenotype (Fernando et al., 2010; Hu et al., 2014).

Our RNA-seq analysis of Ras-transformed zebrafish notochords mimicking activated RTK signaling revealed deregulation of clinically relevant chordoma genes, including *s100a1* and bcl6 family members (Fig. 5). These changes were concomitant with alterations in UPR, ER stress response and ECM pathways (Fig. 5, Fig. S5). Ultimately, the excessive accumulation of ECM collagen sheets around the transformed notochord probably hinders the hyperplastic cells in their expansion (Fig. 6). Hence, RTK-based transformation alone might skew notochord cells into a cellular state that caps their proliferative and invasive potential. Consistent with this notion, we detected deregulated UPR and ER stress pathways, which were accompanied by excessive ECM build up that ultimately entombed individual cells (Fig. 6E,F). Coordinated activation of secretory pathway features with concomitant activation of the UPR is a hallmark of progressive notochord differentiation in medaka and *Xenopus* ([Bibr DMM039545C42][Bibr DMM039545C44], 2009). The prominent secretory activity of differentiating notochord cells, in particular collagen secretion and ECM build up, requires careful fine-tuning by the UPR to prevent apoptosis and aberrant protein accumulation in the ER (Vandewynckel et al., 2013). We therefore hypothesize that the suppression of a UPR and ER stress signature upon RTK/Ras activation in notochord cells prevents their terminal differentiation, keeping them in a proliferative, progenitor-like state that is susceptible to additional oncogenic insults. Therapeutic targeting of RTK signaling could consequently attack a main pathway required for the initial transformation of native notochord cells towards chordoma.

## MATERIALS AND METHODS

### Animal husbandry

Zebrafish (*Danio rerio*) were maintained, collected and staged principally as described (Kimmel et al., 1995; Westerfield, 2007) and in agreement with procedures mandated by the veterinary office of the Universität Zürich and the Canton of Zürich. If not otherwise indicated, embryos up to 5 dpf were raised in temperature-controlled incubators without light cycle at 28°C.

### Vectors, primers and transgenic lines

The *col2a1aR2:KalTA4* driver line was generated as follows: first, the plasmid pE5′-col2a1aR2_minprom was cloned by amplifying the previously described *col2a1aR2 cis*-regulatory element (Dale and Topczewski, 2011) with the primers col2a1aR2 fwd and col2a1aR2 BamHI reverse from zebrafish genomic DNA using the Expand High Fidelity Polymerase System (Roche). The resulting PCR product was then TA-cloned into pENTR5′ (Thermo Fisher Scientific) according to the manufacturer's instructions. The mouse β-Globin minimal promoter (minprom) was amplified from pME-minprom-EGFP (Tamplin et al., 2015) with the primers mouse β-Globin minimal promoter BamHI forward and mouse β-Globin minimal promoter XbaI reverse and cloned into pENTR5′-col2a1aR2. The final plasmid col2a1aR2:KalTA4,myl7:EGFP was assembled using Multisite Gateway Cloning combining pENTR5′-col2a1aR2, pENTR′D-KalTA4 (Distel et al., 2009), p3E_SV40polyA (Tol2kit *#302*) (Kwan et al., 2007) and pDestTol2CG2 (Tol2kit *#395*) (Kwan et al., 2007). Successful recombination was confirmed by restriction digest and Sanger sequencing. Wild-type embryos of the TÜ strain were co-injected with 25 pg of the final plasmid and 25 pg of Tol2 transposase mRNA at the one-cell stage and raised according to standard procedures (Felker and Mosimann, 2016). F2 animals with a single-copy transgene insertion were selected for experimentation. The lines *Tg(-2.7twhh:Gal4)* and *Tg(5xUAS:eGFP-HRASV12)* have been previously described (Burger et al., 2014; Santoriello et al., 2010).

UAS vectors for overexpression of candidate genes were generated using Multisite Gateway Cloning. The ORF of chordoma candidate genes was amplified from human cDNA clones or zebrafish cDNA (see below) using the primers listed in Table S2 and TOPO-cloned into pENTR/D-TOPO (Thermo Fisher Scientific) according to the manufacturer's instructions. Zebrafish *kdr* follows suggested ortholog nomenclature (Bussmann et al., 2007, 2008). Gateway reactions were performed using pENTR5′-4xnr UAS (Akitake et al., 2011), pAF20-3′-2AmCerulean (Kirchgeorg et al., 2018) and pCM326 (pDestTol2CG2,crya:Venus) (Mosimann et al., 2015). *Tg(col2a1aR2:KalTA4,myl7:EGFP)* embryos were injected with 25 pg of the pUAS:candidate together with 25 pg of Tol2 transposase mRNA at the one-cell stage and raised until 5 dpf (Felker and Mosimann, 2016). A list of plasmids used in the transient injection approach is given in Table S3.

### Imaging and staining

Injected embryos were sorted by fluorescence dissecting scope-detectable notochord fluorescence (indicating the presence of the *UAS*-transgene) and imaged at 5 dpf. Animals with strong fluorescence were fixed in 4% paraformaldehyde at 4°C overnight. Subsequently, embryos were embedded in paraffin, sectioned at 5 µm, deparaffinated and stained with Hematoxylin and Eosin (H&E) according to standard protocols. Immunohistochemical studies were performed according to the manufacturer's protocol using anti-pERK (4376, Cell Signaling Technology, 1:500), anti-Cytokeratin (961, Abcam, 1:100), anti-TP53 (GTX128135, GeneTex, 1:200), anti-Tbxta (a gift from Andy Oates, École polytechnique fédérale de Lausanne; Webb et al., 2016) and anti-Brachyury (sc-20109 or sc-166962; Santa Cruz, 1:200). Work on chordoma sections was performed under approval by BASEC-no. 2017-00017 by the Cantonal Ethics Commission Zürich. Live animals were imaged using a Leica M205FA stereo microscope. Histological sections were imaged using a Zeiss Axioscan Z1 Slidescanner using a Plan Apochromat 20× objective.

### Transmission electron microscopy

Wild-type and *HRAS^V12^*-expressing larvae were fixed in 2.5% glutaraldehyde at 8 dpf and processed by transmission electron microscopy (TEM) at the Center for Microscopy and Image Analysis, following standard protocols. Images were acquired using a Philips CM100 transmission electron microscope.

### Notochord isolation and mRNA sequence analysis

Embryos at 8 dpf *Tg(twhh:Gal4)* and *Tg(twhh:Gal4);Tg(UAS:EGFP-HRAS^V12^)* were euthanized using 3% tricaine methanesulfonate (Sigma-Aldrich). Embryos were decapitated and incubated in trypsin-EDTA (Sigma-Aldrich) for 30 min to facilitate tissue dissociation. Notochords were then dissected using tungsten needles and immediately transferred to Trizol-LS (Ambion). We isolated 30-50 notochords per replicate, with a total of three replicates per condition (three wild type, three *HRAS^V12^*). Notochord RNA was extracted following the manufacturer's protocol using Trizol-LS.

RNA-seq reads were mapped to the zebrafish reference genome (*GRCz10*) using STAR (Dobin et al., 2013). Mapped reads were quantified with featureCounts (Liao et al., 2014). EdgeR (Robinson et al., 2010) was then used for differential expression analysis with an FDR cut-off of 5%. Pathway analysis was performed using Ingenuity Pathway Analysis software (https://www.qiagenbioinformatics.com/products/ingenuity-pathway-analysis/) and the Gene Set Enrichment Analysis packages (http://software.broadinstitute.org/gsea/index.jsp) using the log2FC and FDR <0.05.

## Supplementary Material

Supplementary information
