# Supplementary material for: Active receptor tyrosine kinases, but not Brachyury, are sufficient to trigger chordoma in zebrafish
Source: Dis Model Mech. 2019 Jul 16;12(7):dmm039545. doi: 10.1242/dmm.039545 (PMC6679381; doi:10.1242/dmm.039545)
Supplement: Supplementary information [file dmm-12-039545-s1.pdf]

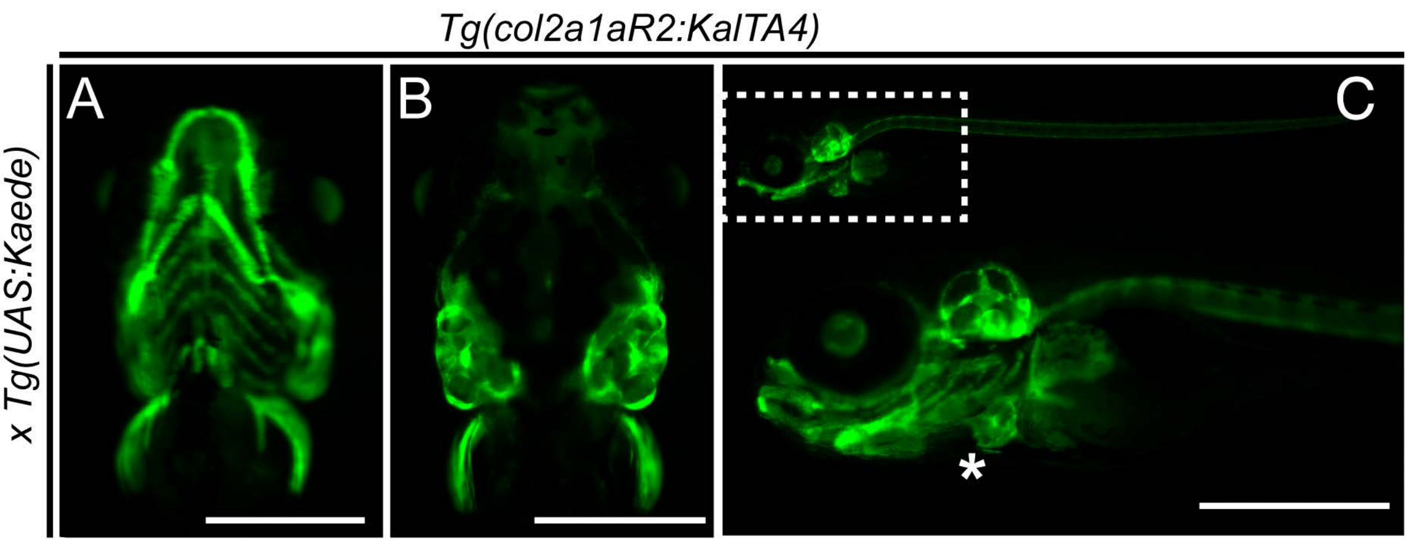

Figure S1, related to Figure 1: Activity of *Tg(col2a1a:KalTA4)* at later developmental stages and adults
(**A-C**) *col2a1aR2:Kal4TA4* activity shown by crossed-in *UAS:Kaede* expression (green fluorescence). (**A**) Ventral view showing the developing jaw cartilage and pectoral fins at 5 dpf. (**B**) Dorsal view showing activity in otic vesicle cartilage and pectoral fins at 5 dpf. (**C**) Lateral view of the craniofacial cartilage expressing *col2a1aR2:KalTA4* at 5 dpf. Asterisk marks the heart labelled with *myl7*:EGFP as transgenesis marker. See also Figure 1.

Disease Models & Mechanisms • Supplementary information

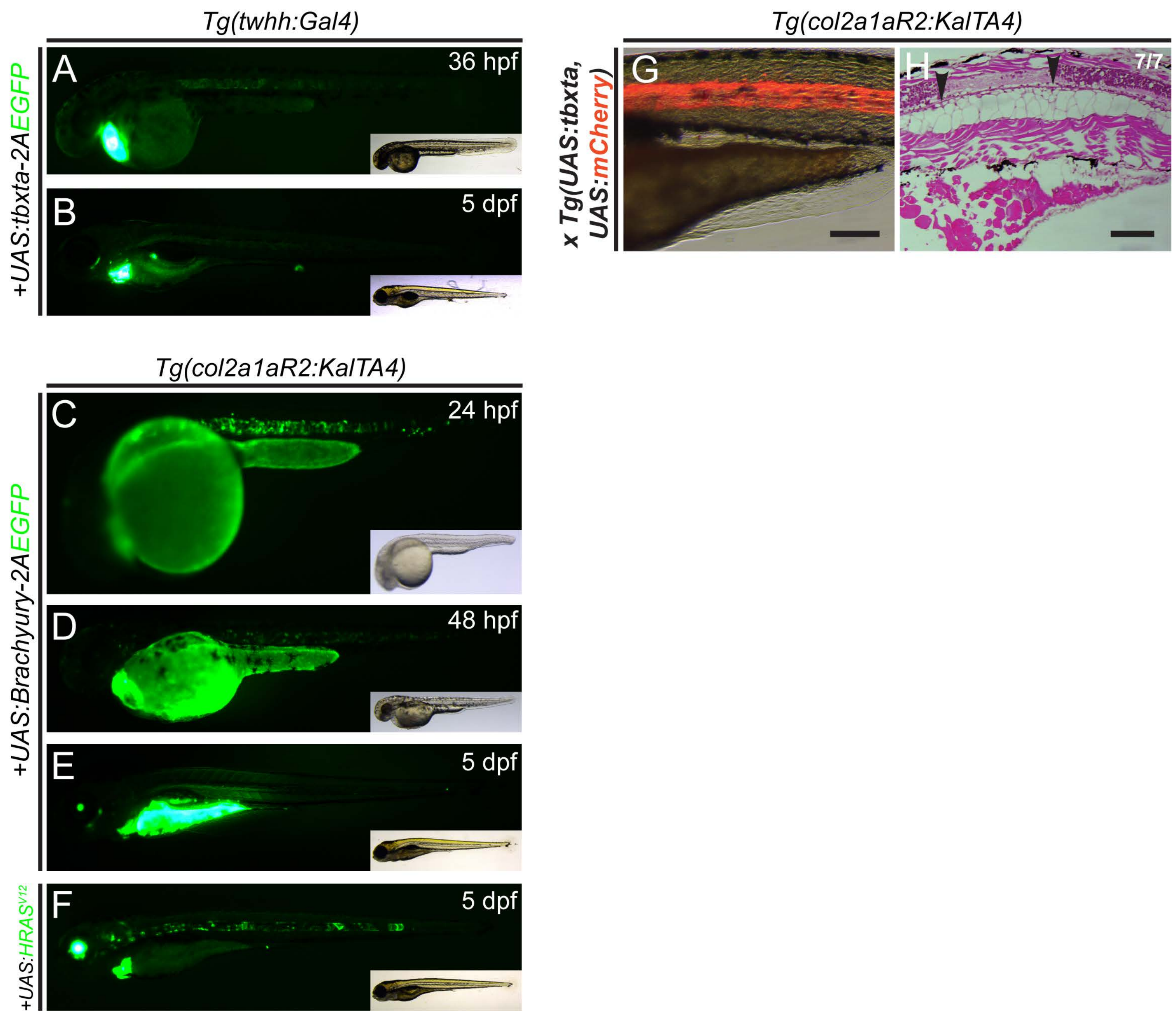

Figure S2, related to Figure 2: Tagged fusion proteins of Brachyury fail to express efficiently *in vivo*. (**A-F**) Lateral views of zebrafish embryos at indicated developmental stages, with EGFP fluorescence (green) in main panels, insets show brightfield for overall embryo morphology. (**A**,**B**) Transgene silencing in *Tg(twhh:Gal4)* with injected *UAS:tbxta-2A-EGFP*; while selectively expressed in the notochord at 36 hpf (n= 43/56), expression greatly reduced at 5 dpf. (**C-F**) Representative embryos transiently expressing *UAS: Brachyury-2A-EGFP* at 24 hpf at detectable levels (**C**), while expression becomes significantly reduced at 48 hpf (**D**) and is completely silenced at 5 dpf (**E**). (**F**) Transiently expressed *UAS:EGFPHRAS*^{V12} at 5 dpf mainly localizes in the notochord and to a minor extent in the otic vesicle. Tumorigenic lesions could only be detected in the notochord. Transgenic markers used are *myl7:EGFP* for *twhh:Gal4* and *col2a1aR2:KalTA4, cryaa:Venus* for injected *UAS*-plasmids. (**G**,**H**) Akin to injected *UAS:tbxta*, stable transgenic *Tg(UAS:tbxta)* overexpression fails to cause any significant notochord defect, with only few areas showing smaller vacuolated cells (arrowheads, **H**). See also Figure 2.

Disease Models & Mechanisms • Supplementary information

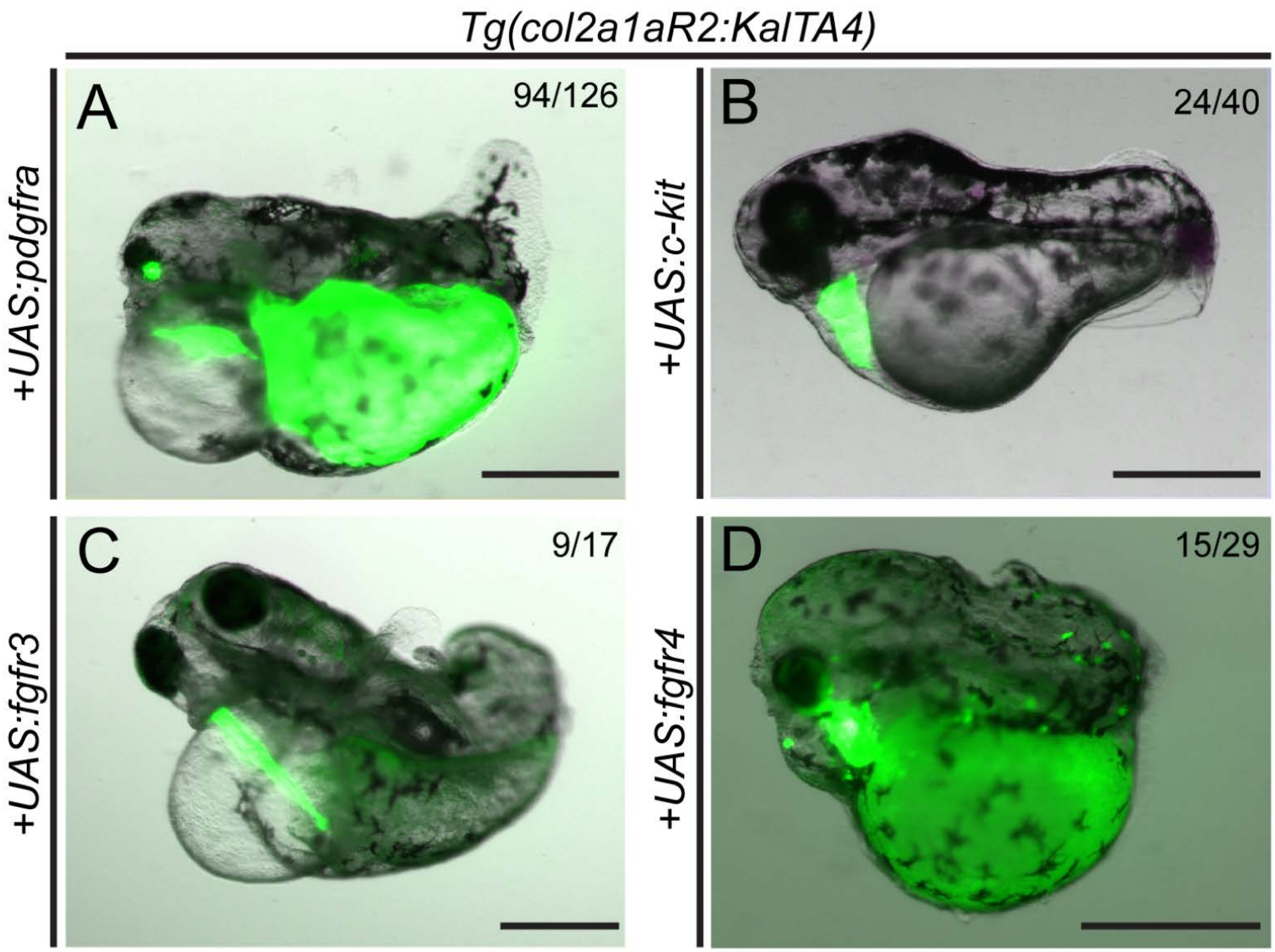

Figure S3, related to Figure 3: Embryo-wide, non-autonomous perturbation of embryo development upon notochord-specific overexpression of individual developmental RTK genes.

(**A-D**) Representative *Tg(col2a1aR2:KalTA4)* embryos injected with individual *UAS* constructs for different chordoma-implicated RTK genes; numbers depict observed phenotype versus phenotypically normal-appearing embryos in a representative experiment (n=3). Notochord specific overexpression of zebrafish *pdgfra* (**A**)*, c-kit* (**B**), *fgfr3* (**C**), and *fgfr4* (**D**) leads to severe body axis truncation and aberrant development of cardiovascular and craniofacial structures (**A, C, D**: 5 dpf, **B**: 3 dpf). Transgenic markers: *myl7:EGFP* for *col2a1aR2:KalTA4, cryaa:Venus* for injected UAS plasmids. Scale: 500 µm. See also Figure 3.

Disease Models & Mechanisms • Supplementary information

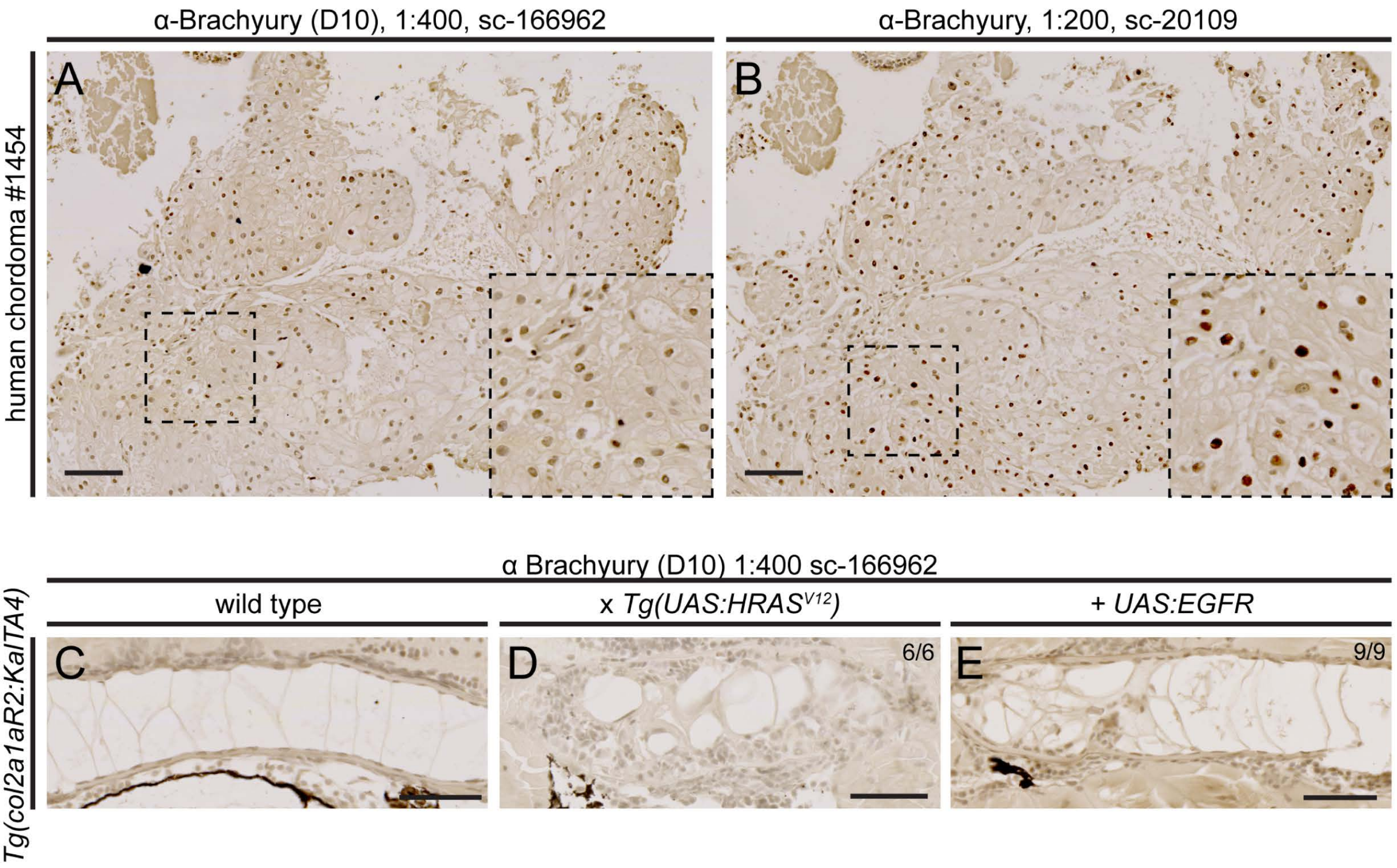

Figure S4, related to Figure 4: Testing of anti-Brachyury antibodies on human chordoma samples and zebrafish notochord sections.

(**A,B**) Human chordoma sections from same tumor (chordoma #1454), stained for Brachyury using α-Brachyury D10 (sc-166962) and a discontinued α-Brachyury antibody (sc-20109) as positive staining control. Both reagents produce a strong nuclear staining. (**C-E**) α-Brachyury D10 fails to stain zebrafish notochord tissue in several overexpression scenarios. See also Figure 4.

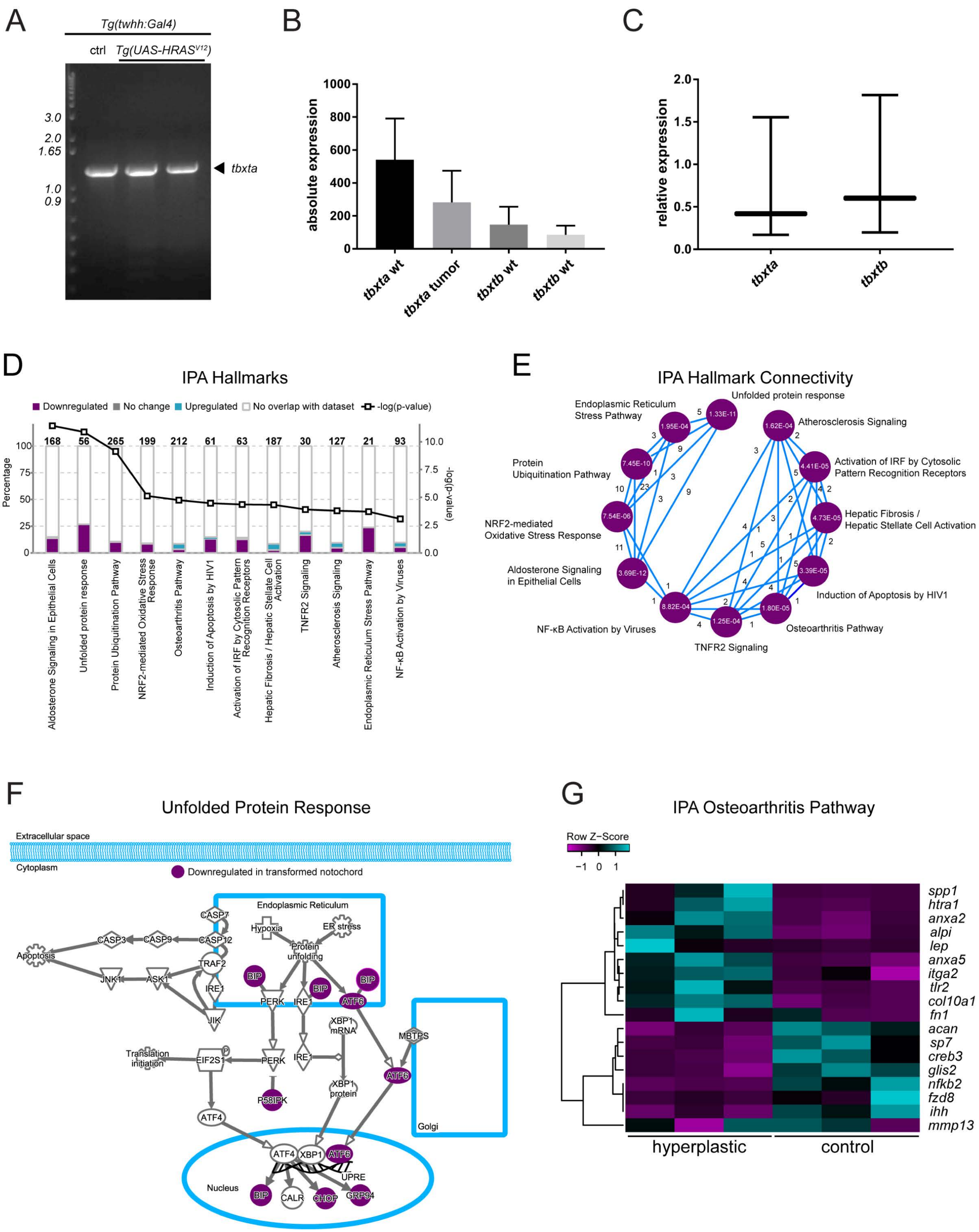

A
Tg(twhh:Gal4)
ctrl
Tg(UAS-HRAS^{V12})
3.0
2.0
1.65
1.0
0.9
tbxta
B
absolute expression
tbxta wt
tbxta tumor
tbxtb wt
tbxtb wt
C
relative expression
tbxta
tbxtb
D
IPA Hallmarks
Downregulated
No change
Upregulated
No overlap with dataset
-log(p-value)
Percentage
Aldosterone Signaling in Epithelial Cells
Unfolded protein response
Protein Ubiquitination Pathway
NRF2-mediated Oxidative Stress Response
Osteoarthritis Pathway
Induction of Apoptosis by HIV1
Activation of IRF by Cytosolic Pattern Recognition Receptors
Hepatic Fibrosis / Hepatic Stellate Cell Activation
TNFR2 Signaling
Atherosclerosis Signaling
Endoplasmic Reticulum Stress Pathway
NF-κB Activation by Viruses
E
IPA Hallmark Connectivity
Unfolded protein response
Endoplasmic Reticulum Stress Pathway
Atherosclerosis Signaling
Protein Ubiquitination Pathway
Activation of IRF by Cytosolic Pattern Recognition Receptors
NRF2-mediated Oxidative Stress Response
Hepatic Fibrosis / Hepatic Stellate Cell Activation
Aldosterone Signaling in Epithelial Cells
Induction of Apoptosis by HIV1
NF-κB Activation by Viruses
Osteoarthritis Pathway
TNFR2 Signaling
F
Unfolded Protein Response
Extracellular space
Cytoplasm
Downregulated in transformed notochord
Endoplasmic Reticulum
Golgi
Nucleus
G
IPA Osteoarthritis Pathway
Row Z−Score
spp1
htra1
anxa2
alpi
lep
anxa5
itga2
tlr2
col10a1
fn1
acan
sp7
creb3
glis2
nfkb2
fzd8
ihh
mmp13
hyperplastic
control

Figure S5, related to Figure 5: Transcriptome analysis of wildtype versus transformed zebrafish notochords reveals maintained expression of *Brachyury* genes and deregulated pathways.

(**A-C**) Zebrafish *Brachyury* genes *tbxta* and *tbxtb* genes remain expressed beyond early development. (**A**) RT-PCR of isolated 8 dpf wildtype and *HRAS*^{V12}-expressing notochords (two independent samples), respectively, confirms ongoing expression of *tbxta*. (**B**,**C**) Absolute (number of reads) and relative levels of *tbxta* and *tbxtb* expression in wildtype and *HRAS*^{V12}-expressing notochords. Compared to *tbxta, tbxtb* is also expressed but at lower levels. Differences between wildtype versus *HRAS*^{V12}-expressing samples are not significant. (**D**) Ingenuity Pathway Analyis (IPA) output for the twelve most-significantly enriched pathways deregulated in transformed notochords; note repeated association with protein stress, unfolded protein response (UPR), and secretion of the enriched pathways. (**E**) Circle plot of main uncovered processes via IPA, with blue lines connecting Hallmark sets that share genes (numbers associated with blue lines); numbers inside Hallmark circles represent p-values. (**F**) Schematic of the unfolded protein response (URP) pathway, with components found downregulated in transformed zebrafish notochords shown in color. (**G**) Expression heat map of the IPA hallmark Osteoarthritis Pathway, encompassing key regulators of bone formation that are de-regulated between control versus hyperplastic zebrafish notochords. See also Figure 5.

Disease Models & Mechanisms • Supplementary information

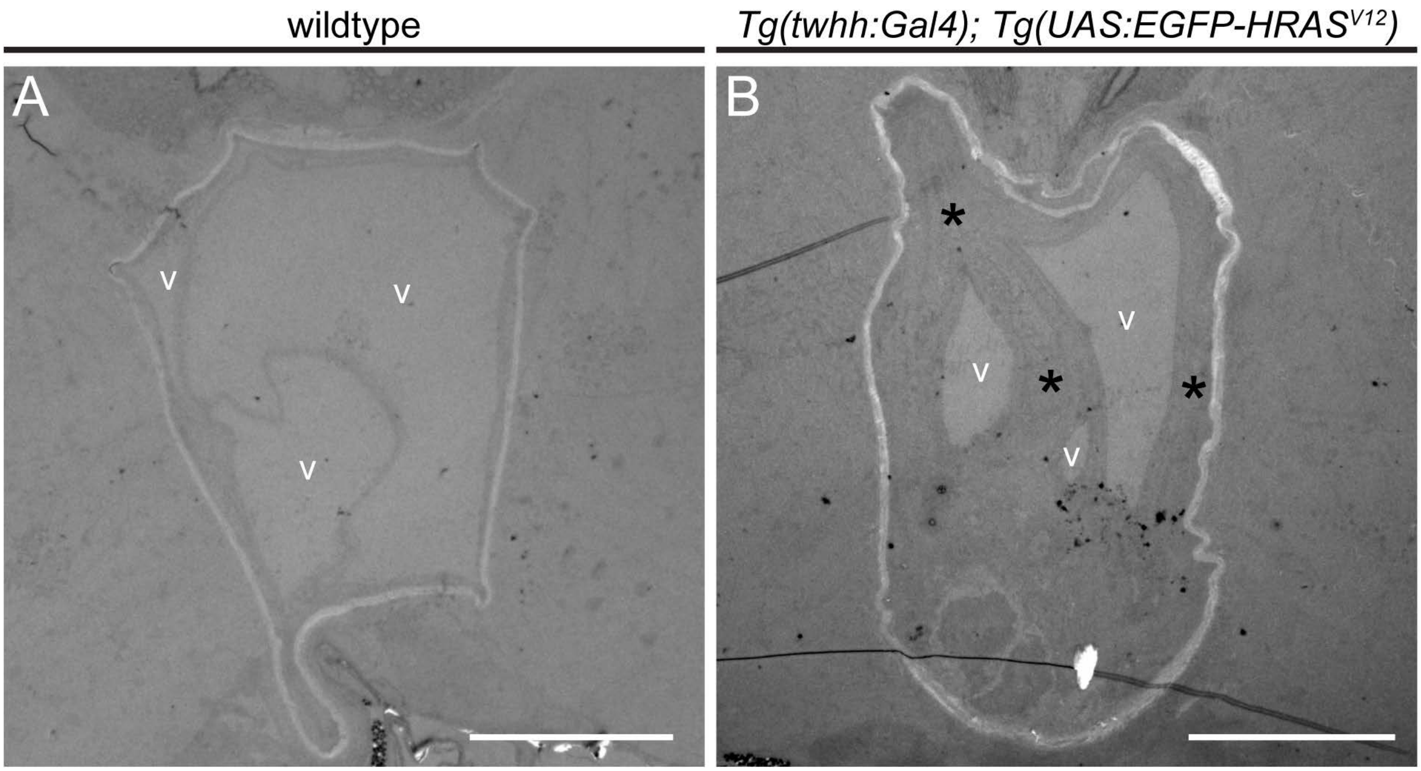

Figure S6, related to Figure 6: Overview of notochord sections used for EM

(**A,B**) Transverse section through a wildtype (**A**) and a representative *HRAS*^{V12}-expressing notochord (**B**). Vacuolated cells (V) make up the majority of the notochord volume in wildtype, but are are markedly smaller upon transformation due to overproliferating sheath cells converging towards the center of the notochord (asterisks in **B**). Note the light band of ECM surrounding the notochords. Deformation of the natively circular notochord shape results from fixation procedures for EM. See also Figure 6. Scale bars: 25 μm.

Table S1, related to Figure 5, S5: RNA-seq results

Compiled RNA-seq results depicted in Figures 5 and S5, also deposited as ArrayExpress accession E-MTAB-7349.

Click here to Download Table S1

Table S2: Primers used in this study.

**Primer name**	**Sequence (5'-3'; restriction sites/features underlined)**
Mouse beta-Globin minimal promoter BamHI forward	*AAAGGATCCCCATGGCCAATCTGCTCAGA*
Mouse beta-Globin minimal promoter XbaI reverse	*AAATCTAGACCATGGGATGTCTGTTTCTG*
c-kit forward	*CACCATGGAATATCACTGCGTTCT*
c-kit no stop reverse	*GACTACAGGGTGACTTGGAC*
col2a1a R2 forward	*CCTCTGACACCTGATGCCAATTGC*
col2a1a R2 BamHI reverse	*AAAGGATCCAGGGATATGTGTATGTGTGT*
fgfr3 forward	*CACCATGGTCCCACTCTGTCTCCT*
fgfr3 no stop reverse	*TGTTCGTATGACCCCGTTGC*
fgfr4 forward	*CACCATGTTGAGCATCTTAAAGGT*
fgfr4 no stop reverse	*TCGCATTGTCGTCTTTAGGT*
KalTA4 forward	*CACCATGAAACTGCTCTCATCCAT*
KalTA4 reverse	*TTAGTTACCCGGGAGGATGT*
kdr forward	*CACCATGGCAAAGACATCTTATGC*
kdr no stop reverse	*AACTGGAGGTGCACTATAAC*
tbxta forward	*CACCATGTCTGCCTCAAGTCCCGA*
tbxta no stop reverse	*GTAGCTCTGAGCCACAGGCG*
tbxta stop reverse	*TCAGTAGCTCTGAGCCACAG*
tbxtb forward	*CACCATGGACTCCGGTGACTGTCCT*
tbxtb no stop reverse	*TAAGGATGGTGGTGCCACATG*
pdgfra forward	*CACCATGTTCCCGGTGCTGCCACA*
pdgfra no stop reverse	*CAGGAAGCTGTCCTCCACCA*
stat3 EcoRI forward	*AAAGAATTCATGGCCCAGTGGAATCAGTT*
stat3 XhoI reverse	*TTCTCGAGCTAAGCATTTCGGCAGGTGT*
T/Brachyury forward	*CACCATGAGCTCCCCTGGCACCGA*
T/Brachyury no stop reverse	*CATGGAAGGTGGCGACACAG*
T/Brachyury stop reverse	*TCACATGGAAGGTGGCGACA*

Table S3: List of plasmids used in the transient injection approach

Plasmid name	Overexpressed ORF
UAS:EGFR-2AEGFP	*EGFR* (human), fusion protein with *2A-EGFP* ORF
UAS:fgfr3-2AEGFP	*fgfr3* (zebrafish), fusion protein with *2A-EGFP* ORF
UAS:fgfr4-2AEGFP	*fgfr4* (zebrafish), fusion protein with *2A-EGFP* ORF
UAS:EGFP-HRAS^{V12}	*HRAS^{G12V}* (human), direct fusion with *EGFP* ORF
UAS:kdr-2Acerulean (vegfr2)	*kdr/vegfr2* (zebrafish), fusion protein with *2A-EGFP* ORF
UAS:c-kit-2AEGFP	*kita/c-kit* (zebrafish), fusion protein with *2A-EGFP* ORF
UAS:tbxta	*tbxta* (zebrafish)
UAS:tbxta-2AEGFP	*tbxta* (zebrafish), fusion protein with *2A-EGFP* ORF
UAS:tbxta-VP16	Zebrafish *tbxta aa1-232+VP16* transactivation domain
UAS:tbxtb	*tbxtb* (zebrafish)
UAS:tbxtb-2AEGFP	*tbxtb* (zebrafish), fusion protein with *2A-EGFP* ORF
UAS:pdgfra-2AEGFP	*pdgfra* (zebrafish), fusion protein with *2A-EGFP* ORF
UAS:EGFP-rheb	*rheb* (zebrafish)
UAS:EGFP-stat3	*stat3* (zebrafish)
UAS:Brachyury	*Brachyury* (human)
UAS:Brachyury-2AEGFP	*Brachyury* (human), fusion protein with *2A-EGFP* ORF
UAS:EGFP	*EGFP*

Disease Models & Mechanisms • Supplementary information
